# Applications of Solid-State NMR Spectroscopy for the Study of Lipid Membranes with Polyphilic Guest (Macro)Molecules

**DOI:** 10.3390/polym8120439

**Published:** 2016-12-16

**Authors:** Ruth Bärenwald, Anja Achilles, Frank Lange, Tiago Mendes Ferreira, Kay Saalwächter

**Affiliations:** Institut für Physik—NMR, Martin-Luther-Universität Halle-Wittenberg, D-06120 Halle, Germany

**Keywords:** lipid membranes, lipid–polymer composites, solid-state NMR spectroscopy, order parameter

## Abstract

The incorporation of polymers or smaller complex molecules into lipid membranes allows for property modifications or the introduction of new functional elements. The corresponding molecular-scale details, such as changes in dynamics or features of potential supramolecular structures, can be studied by a variety of solid-state NMR techniques. Here, we review various approaches to characterizing the structure and dynamics of the guest molecules as well as the lipid phase structure and dynamics by different high-resolution magic-angle spinning proton and ^13^C NMR experiments as well as static ^31^P NMR experiments. Special emphasis is placed upon the incorporation of novel synthetic polyphilic molecules such as shape-persistent T- and X-shaped molecules as well as di- and tri-block copolymers. Most of the systems studied feature dynamic heterogeneities, for instance those arising from the coexistence of different phases; possibilities for a quantitative assessment are of particular concern.

## 1. Introduction

Biological membranes play an important role in living matter. They form boundaries of cells and organelles within the cell and they are the site of many biochemical events. They consist of a complex heterogeneous and dynamic mixture of lipids, proteins and other components such as carbohydrates, and cholesterol [1,2]. The lipid molecules mostly organize in a bilayer building a permeability barrier. To this basic lipid matrix the membrane proteins are bound in different forms providing various functions such as stabilizing the membrane, acting as receptors or transporters for enzymes, or forming pores or channels to transport ions through the bilayer [3,4].

The particular biological function of a membrane is determined by its composition and the organization of its components. Of major relevance for membranes is the formation of domains enriched with certain lipids or proteins which provide specific functions. In biomembranes, there exist various kinds of domains with different chemical compositions and sizes [2]. Namely, within the last two decades, the formation and function of so-called lipid rafts in cells—small and highly mobile membrane domains enriched in sterol and sphingolipid—has become a key concept in membrane science [2,5,6].

In general, the study of cell membranes on the molecular-scale is a major research goal. However, as biological membranes are very complex, often model membranes just consisting of phospholipids are used for simplification. Frequently, phosphatidylcholines are used for investigations. They consist of a choline headgroup, a glycerol backbone and two hydrocarbon chains which can be saturated in dimyristoylphosphatidylcholine (DMPC) or dipalmitoylphosphatidylcholine (DPPC) or unsaturated, for example in dioleoylphosphatidylcholine (DOPC). Depending on the temperature, such lipid bilayer samples can be found in the gel or the liquid-crystalline phase and in multicomponent phospholipid systems, species immiscibility and phase separation can occur.

Significant research activities are devoted to synthetic polymers or other complex molecules that can be embedded in lipid bilayers or attached to the surfaces and consequently change membrane properties or alter phase transitions. Also, phase separation and domain formation can occur as it does in biological membranes. Specific molecules can form pores or channels in bilayers [7], and can act as artificial ion [8] or glucose [9] transporters. A prominent example of molecules interacting with lipid bilayers are amphiphilic block copolymers, such as the well-known Pluronics, which consist of hydrophilic poly(ethylene oxide) (PEO) end blocks and a hydrophobic poly(propylene oxide) (PPO) middle block. They can act as drug-delivery system [10], help to overcome drug resistance [11], or seal damaged membranes [12,13].

Solid-state nuclear magnetic resonance (SSNMR) spectroscopy provides probably the most powerful set of experimental techniques suited to study structural aspects, orientational order and the dynamics of membrane components, and the molecular basis of interactions with guest molecules [14]. This review presents an overview of a variety of NMR techniques that are relevant for the study of guest (macro)molecules in interaction with lipid membranes, and illustrates these with results obtained within the Research Unit FOR1145 funded by the German Research Foundation (Deutsche Forschungsgemeinschaft, DFG) for sample systems containing “*polyphilic*” block copolymers and bolapolyphiles. The molecular structure of the polyphilic guest molecules was varied systematically to develop a detailed understanding of the interaction with the lipid molecules and the properties of the membrane as a whole. In the following, the scope of the FOR1145 and some key insights shall be presented.

To first explain the term “*polyphilic*”, we remind that amphiphilic molecules are particularly well suited for the interaction with lipid bilayers, as the lipophilic molecule part can be incorporated into the lipid acyl chain region while hydrophilic parts can interact with the lipid headgroup or stay in the surrounding water. These interactions can now be tuned further by using polyphilic molecules, which represent an extension of the amphiphilic molecules as they contain special units such as mesogenic (liquid crystal forming) or fluorinated parts in addition to the amphiphilic moiety. In interaction with a membrane, these additional parts can for instance cause self-assembly of the guest molecules or the formation of a composite structure with lipids which show a similar behavior as in lipid rafts, or in the liquid-ordered phase of phospholipid-cholesterol systems.

**Figure 1:**
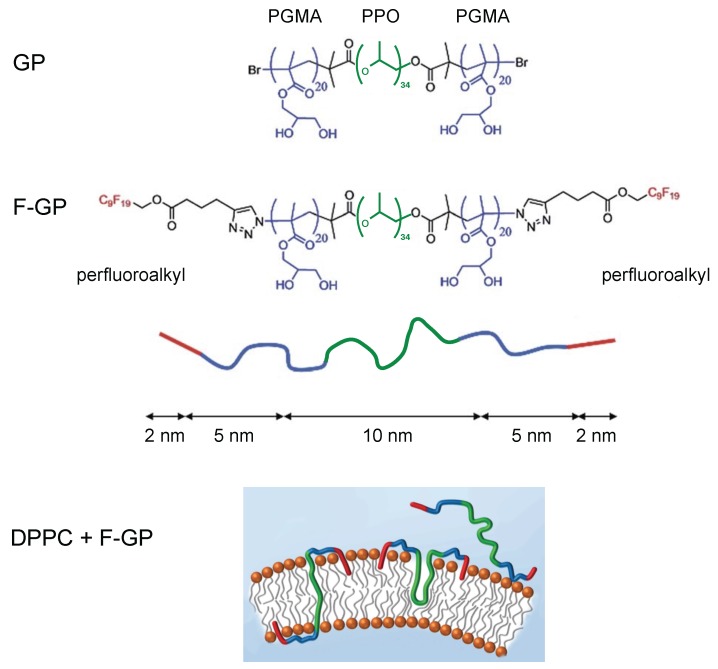
Molecular structure of the triblock copolymer PGMA_20_-PPO_34_-PGMA_20_ (GP) and the pentablock copolymer F_9_-PGMA_20_-PPO_34_-PGMA_20_-F_9_ (F-GP). The approximate contour length of the hydrophobic (green), hydrophilic (blue) and fluorophilic (red) block is given. The different possibilities of the pentablock copolymer F-GP to interact with the lipid bilayer are shown schematically. Figure adapted from ref. [16] published by the Royal Society of Chemistry.

Extensive research was conducted on sample systems of Pluronics in mixture with lipid bilayers. To modify the lipid–polymer interaction, a new amphiphilic triblock copolymer was created by replacing the hydrophilic PEO-blocks by poly(glycerol monomethacrylate) (PGMA) blocks [15]. PGMA has a higher propensity to act as a hydrogen bond donor and acceptor, so that it should interact differently with the lipid head group. The PGMA monomer is also more bulky and could therefore cause a stronger disturbance of the membrane [16]. Specifically, the triblock copolymer PGMA_20_-PPO_34_-PGMA_20_ (GP) (see Figure 1) was investigated. The PPO block of this polymer is long enough to possibly span a bilayer.

Furthermore, a polyphilic pentablock copolymer was created by attaching additional perfluoroalkyl chains to both ends of the PGMA blocks. In Figure 1, the structure of F_9_-PGMA_20_-PPO_34_-PGMA_20_-F_9_ (F-GP) is shown [17]. The fluorinated chains are hydrophobic, but not lipophilic. Instead, they are fluorophilic and separate into an own phase [16]. The incorporation of these block copolymers in lipid bilayers was proven and investigated in more detail by monolayer studies using infrared reflection absorption spectroscopy coupled with Brewster angle microscopy and surface pressure measurements [18,19] and by differential scanning calorimetry (DSC) [16].

**Figure 2:**
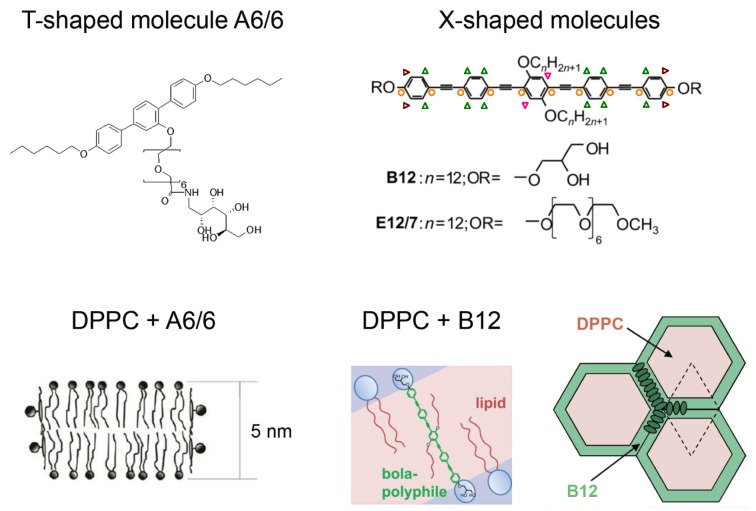
Chemical structures of various bolapolyphiles (**top**); and schemes of their modes of interaction with lipid bilayers (**bottom**). Assigned groups of ^13^C resonances of the aromatic core of the X-shaped molecules are marked by different symbols (circles and triangles). Figure adapted from refs. [21,22,23] with permissions from the publishers. Copyrights 2015 John Wiley& Sons and 2016 Americal Chemical Society.

Bolapolyphilic molecules (BPs) are another big group of substances that were investigated in interaction with model lipid bilayers. One example is the T-shaped facial polyphilic molecule A6/6 [20,21], the structure of which is shown in Figure 2. The molecule consists of a hydrophobic rodlike terphenyl core with two predominantly hydrophobic and flexible terminal hexyloxy alkyl chains at both ends. A hydrophilic lateral oligo(oxyethylene) chain terminated by a carbohydrate derivative is connected to the central phenyl ring of the core. A6/6 disrupts the membrane and destroys vesicles. Instead, they form planar bilayer patches of hexagonal symmetry (bicelles) with the A6/6 molecules bordering the membrane edges [21], see Figure 2.

Figure 2 also shows the molecular structures of X-shaped triphilic molecules which are another example of bolapolyphiles that can interact with lipid bilayers [22]. These molecules consist of a stiff hydrophobic π-conjugated phenylene–ethinylene backbone of about 3 nm length, which roughly matches the hydrophobic thickness of a DPPC bilayer. The rigid core is terminated by two identical hydrophilic head groups. Additionally, two flexible hydrophobic alkyl chains are connected to the central phenyl ring. In interaction with lipid bilayers, these X-shaped bolapolyphiles form supramolecular structures that can be tuned by structural variations of the molecules, such as different hydrophilic head groups or different lengths of the side chains [22].

The bolapolyphile B12 has glycerol head groups and side chains with 12 carbons. Giant unilamellar vesicles (GUVs) formed of DPPC in mixture with B12 were investigated by fluorescence microscopy revealing domains of snowflake-like structure with six-fold symmetry and dendritic branches pointing to a regular packing structure of B12 and lipid molecules [22]. From orientation-dependent (polarized) fluorescence measurements, transmembrane orientation of B12 was deduced. As a result of further X-ray diffraction and NMR measurements, self-organization of B12 into honeycomb lattices was suggested for the mixture with DPPC [22], see Figure 2 for a schematic structure. The honeycomb walls are formed by the rod-like π–π-stacked backbones and its cells accommodate the alkyl side chains as well as confined lipids. The glycerol headgroups of B12 are aligned along the upper and lower edges of the honeycombs. DOPC and B12 on the other hand form a homogeneous mixture [23].

The bolapolyphile E12/7, also shown in Figure 2, is another X-shaped molecule that was investigated in detail. E12/7 has an oligo(ethylene oxide) (EO_7_) headgroup which is incapable of acting as a hydrogen-bond donor. Therefore, E12/7 interacts significantly differently with DPPC as compared to B12. Fluorescence measurements confirmed the approximately transmembrane orientation of E12/7, but domains on the micrometer scale were not visible [22]. Instead, E12/7 forms a rather rigid self-assembled filamentous structure on the nanometer scale, which is randomly distributed in the lipid matrix and does not involve lipid molecules [23].

In the following, some basic concepts of SSNMR spectroscopy will be summarized. We then turn to, first, applications of high-resolution techniques based upon magic-angle spinning (MAS), allowing for a moiety-based assessment of structural and dynamic aspects by resolving ^1^H and ^13^C chemical shifts. An extension to higher spectral dimensions allows one to probe dynamic order parameters associated with specific ^13^C–^1^H or ^1^H–^1^H bond orientations, which is the subject of the next section covering so-called proton- and carbon-detected separated local-field (SLF) spectroscopy and proton double-quantum (DQ) NMR, respectively. These techniques have come to rival the more established static ^2^H NMR [24], which requires specific isotope labeling and is less resolved unless single-site labeling is applied. Finally, we illustrate the use of static ^31^P NMR, which provides a probe of slow (μs to ms) headgroup motions reflecting either lipid diffusion along a curved membrane or motions of the membrane itself, such as undulations.

## 2. NMR Basics and Applications to Lipid Systems

### 2.1. Interactions in SSNMR

The resonance frequency of a spin in a magnetic field is mainly determined by the Zeeman effect, providing the Larmor frequency $\omega_{0}$, with important correction terms treated within first-order perturbation theory. These comprise internal interactions of the spin with its electronic surrounding or other spins. As the basis for NMR experiments, these interactions provide the relevant molecular-level information. The most important interactions for a spin-1/2 system in a solid sample are the chemical shift and the dipolar interaction.

The chemical shift arises from partial shielding of a nucleus from the external magnetic field by secondary magnetic fields caused by moving electrons. Due to the chemical shift, the resonance frequency in the NMR spectrum is sensitive to the chemical surrounding of a nucleus, such as the directly bonded or other neighboring atoms or interactions with surrounding molecules. For fast-tumbling molecules in solution, there is no orientation dependence and each chemically different nucleus in general gives rise to a separate resonance line with a characteristic isotropic chemical shift. In solids however, we need to consider the chemical shift anisotropy (CSA) which can be described in terms of a cartesian chemical-shift tensor. The resonance frequency in the spectrum can be calculated from

(1) $$\omega_{\text{CS}}=\omega_{0}\left(\sigma_{\text{iso}}+\frac{\delta }{2}\left(3\cos^{2}\theta -1+\eta \sin^{2}\theta \cos2\varphi \right)\right)$$

where $\sigma_{\text{iso}}$ is the isotropic chemical shift, *η* the asymmetry parameter and *δ* the anisotropy parameter. The polar angles *φ* and *θ* transfer the chemical shift tensor from its principle axis system to the laboratory frame defined by $B_{0}$. As all orientations occur in a powder sample, we need to average over all angles. The result is a broad spectrum with the typical powder pattern form, for a specific example see Figure 18b below. For lipid molecules in a fluid bilayer, the CSA tensor shows axial symmetry due to the fast reorientation of the lipids about their long axis. Therefore, the asymmetry parameter *η* is zero and Equation (1) simplifies to

(2) $$\omega_{\text{CS}}=\omega_{0}\left(\sigma_{\text{iso}}+\langle \delta \rangle \;P_{2}\left(\cos\beta \right)\right),$$

where $P_{2}\left(\cos\beta \right)=\frac{1}{2}\left(3\cos^{2}\beta -1\right)$ is the second Legendre polynomial and 〈δ〉 is the anisotropy parameter of the time-averaged CSA tensor that is now oriented along the lamellar normal. The angle *β* thus describes the orientation of the latter with respect to $B_{0}$. In this case, the resulting powder spectrum is characterized by a high field peak and a low field shoulder for lamellar normal orientations perpendicular and parallel to the magnetic field, respectively (see Figure 18b below).

Another main reason for line broadening in SSNMR is the dipole–dipole coupling which arises from the interacting magnetic moments of the nuclear spins. The dipolar coupling between two spins depends on the length *r* and orientation *θ* of their connecting vector. The effect on the spectrum can be calculated from

(3) $$\omega \left(\beta \right)=\omega_{\text{CS}}\pm D\;P_{2}\left(\cos\theta \right),$$

where $D\propto 1/r^{3}$ is the dipole–dipole coupling constant. In organic solids, strong multiple homonuclear couplings between the ^1^H are present (for which one has to replace *D* by $\frac{3}{2}D$). The respective spectrum of a rigid (immobile) multispin system shows a broad nearly Gaussian-shaped line with a width of several tens of kHz [25].

Scalar or J-couplings (also called indirect dipole–dipole coupling) are not very relevant for SSNMR, but they play an important role in solution NMR. This interaction is mediated by the electrons of the chemical bonds connecting the two nuclear spins.

### 2.2. High-Resolution SSNMR

SSNMR has to deal with broad spectral lines due to strong anisotropic interactions such as the dipolar coupling, the chemical shielding or quadrupolar interactions. However, there are possibilities to obtain spectral resolution, for example by use of oriented samples. For lipid systems, orientation can be achieved by stacking the bilayers onto thin glass plates or through magnetic alignment in solution [26]. However, in many cases these methods are unfavorable due to experimental problems such as low signal, or in the case of glass plates a poor mechanical stability or dehydration.

Probably the most relevant possibility to enhance spectral resolution is magic-angle spinning (MAS). Thereby, the sample is contained in a cylindrical NMR rotor which rapidly rotates about an axis inclined by the magic-angle of $\beta_{m}=$ 54.74° with respect to the static magnetic field. In this way, anisotropic interactions which exhibit a $P_{2}\left(\cos\theta \right)$ dependence can be averaged partially or completely, since ${\langle P_{2}\left(\cos\theta \right)\rangle }_{t}$ = 0, exploiting addition theorems of spherical harmonics and that $P_{2}\left(\cos\beta_{m}\right)$ = 0. When the spinning frequency is about three times or more greater than the anisotropic interaction, then the powder pattern is reduced to a single line at the isotropic value. In this way, chemical-shift resolution is regained, comparable to what is common in solution-state NMR. When the spinning speed is less than or comparable with the size of the anisotropic interaction, then a sideband spectrum is observed with a central resonance at the isotropic value and sidebands spaced with a distance equal to the rotor frequency. For the investigation of lipid samples, moderate spinning frequencies of about 5 to 10 kHz are usually sufficient.

Although MAS allows for chemical resolution, it is also combined with the loss of precious information contained in the anisotropic interactions such as the CSA or the dipolar coupling. However, the information of the underlying anisotropic interaction can be extracted either from the spinning sidebands observed, or by other, more quantitative methods, so-called recoupling pulse sequences. These yield a 2D spectrum containing the isotropic chemical shift site specificity in one dimension, and the structural or dynamic information from the recovered anisotropic interaction in the second dimension.

The resonances of often lowly abundant, low-*γ* nuclei such as ^13^C or ^31^P in SSNMR are usually broadened due to strong heteronuclear dipolar interactions with surrounding ^1^H nuclei. MAS at moderate spinning speeds cannot average these interactions completely. Therefore, heteronuclear decoupling, i.e., radio frequency (rf) irradiation on the ^1^H channel, needs to be applied during the signal acquisition. Originally, just continuous-wave (CW) decoupling was employed. Yet, by now there exist many different phase-modulated pulse schemes which provide a better decoupling efficiency with reduced rf field strength, therefore causing less sample heating. Standard decoupling schemes often used for lipid samples are the TPPM [27] and SPINAL64 [28] sequences.

### 2.3. Overview of SSNMR Applications to Lipid Membranes

SSNMR experiments using probes such as ^1^H, ^2^H, ^13^C or ^31^P can be applied to characterize structural and dynamic properties of lipid membrane components. Straightforward observables such as line widths and intensities in simple ^1^H MAS spectra provide valuable information about molecular dynamics. Chemical shifts in ^13^C spectra contain structural information and the comparison of spectra recorded with different excitation schemes yields dynamical information. This will be the subject of the next section. For example, a MAS NOESY experiment (see below) allows for the localization of mobile guest molecules in fluid bilayers. The arrangement of more rigid molecules in a bilayer can be investigated using ^1^H spin diffusion experiments, which probe the transfer of magnetization through space via dipolar couplings. Recently, Huster, Yao and Hong were able to detect membrane-embedded domains and surface-bound residues of a protein in a bilayer by analyzing ^1^H spin diffusion build up curves. This method relies on the mobility difference between the mobile lipid chains, where spin diffusion is slow, and rigid proteins, allowing for a fast magnetization transfer [29]. A new variant of the technique applicable under the static-solid condition of a lipid gel phase has been introduced more recently [30].

A variety of molecular motions occur in membrane systems over a broad time window from picoseconds to hours. In general, a stochastic rotational or translational motion can be described by a correlation time which is a measure for how long it takes to randomize an atomic position or the orientation of an internuclear vector. Molecular motions in a lipid bilayer with correlation times of several picoseconds to nanoseconds, we define as fast dynamics. This includes librational motions of the bonds, gauche/trans isomerization, diffusional reorientation of lipids about their long axis and wobbling of lipid molecules. For investigating these fast motions, the spin-lattice relaxation time $T_{1}$ and its rotating-frame analogue $T_{1\rho }$ measured during radio-frequency irradiation of suitable nuclei can be used. $T_{1}$ is sensitive to motions on the timescale of the inverse Larmor frequency and contains information about motional geometry and correlation times [31], and $T_{1\rho }$ provides a complement towards slower motions on the timescale of μs. A joint analysis of both quantities can be employed to reliably extract the effective correlation time and the effective amplitude in the fast-motion regime [32].

The determination of dipole–dipole couplings is another possibility to investigate amplitude and geometry of fast but anisotropic motions with correlation times much smaller than the inverse coupling constant of a few kHz. The extent of anisotropy is quantified in terms of a dynamic order parameter that is proportional to the motion-averaged, residual dipolar coupling (RDC). ^13^C–^1^H RDCs can be determined from dipolar-shift correlation (DIPSHIFT) dephasing curves or the R-PDLF experiment, which both belong to the class of separated local-field (SLF) experiments, to be explained in detail below. Double-quantum (DQ) experiments provide analogous ^1^H–^1^H RDCs. These experiments have, to some degree, already replaced the classical static ^2^H NMR experiments focusing on (residual) quadrupolar couplings [24], which require specific isotope labeling and are not covered in detail in this review. Note that, recently, higher-dimensional MAS experiments have been developed for globally ^2^H-labeled biomolecular solids [33,34], allowing for site-specific detection of quadrupolar powder patterns and thus order parameters. Future applications to lipid samples will certainly benefit from the increased accuracy related to the larger quadrupolar (as compared to dipolar) coupling constant.

Intermediate to slow molecular motions occur in the microsecond to lower millisecond range. An example of motions on this timescale is the reorientation of lipid molecules due bilayer undulations and lateral diffusion of the lipid molecules, which can, for example, be investigated by the help of transverse relaxation times $T_{2}$ of ^31^P [35,36] or ^2^H [36,37]. The former is addressed in the last section of this review.

Actual diffusion coefficients of the lipid molecules or the inter-membrane water can be measured by pulsed-field-gradient NMR [38,39,40], which is beyond the scope of this review. We also exclude the study of “ultraslow” motions, such as the tumbling of a whole vesicle or flip-flop motions of the lipids between the two leaflets of the bilayer. These occur on a timescale of many milliseconds up to hours, and can be studied by exchange NMR techniques [40,41] and real-time NMR.

### 2.4. Experimental Aspects

The preparation of lipid model membranes for the SSNMR investigations covered herein is rather straightforward. Usually, an amount of about 10–30 mg lipid is needed to ensure a reasonable signal-to-noise ratio also for the less sensitive ^13^C measurements at natural abundance. Lipid/polyphile mixtures can be prepared by just co-dissolving the molecules in a chloroform/methanol solution, then evaporating the solvent and afterwards rehydrating the sample with D_2_O. This usually results in the formation of micrometer-sized multilamellar vesicles (MLVs), which are rather concentrated in lipids. A water content of about 50 wt % is enough to fully hydrate the lipid molecules which means that structure and dynamics of the lipid bilayer are nearly independent of water content, hence adding more water would not change the spectra or other results.

MLV samples are soft solids in which the slow overall tumbling or lateral diffusion along the low-curvature vesicles fail to average out dipolar interactions and the chemical shift anisotropy. Therefore, MAS is required to obtain high-resolution spectra. Furthermore, a sufficiently precise temperature regulation is necessary for meaningful results, but is, in practice, challenged by different factors. Significant warming from sample rotation and radiofrequency (rf) irradiation must be considered in the temperature calibration [42] and the use of long recycle delays, respectively [43]. Using the well-known phase transition temperatures of pure lipid samples, the temperature calibration can be tested.

## 3. High-Resolution SSNMR Applications to Lipid Membranes

### 3.1. Quantitative ^1^H MAS Spectra

For lipid sample systems, well resolved ^1^H spectra can be acquired using moderate MAS spinning frequencies of 5–10 kHz [44,45,46]. These spectra yield direct information about chemical structure and even, though qualitatively, about molecular dynamics. For example the gel to liquid-crystalline (fluid) phase transition can be investigated. To acquire quantitative ^1^H spectra, several experimental conditions need to be fulfilled, e.g., the use of weight-controlled samples, a recycle delay much larger than the $T_{1}$ relaxation time, and Curie correction for the temperature dependence.

**Figure 3:**
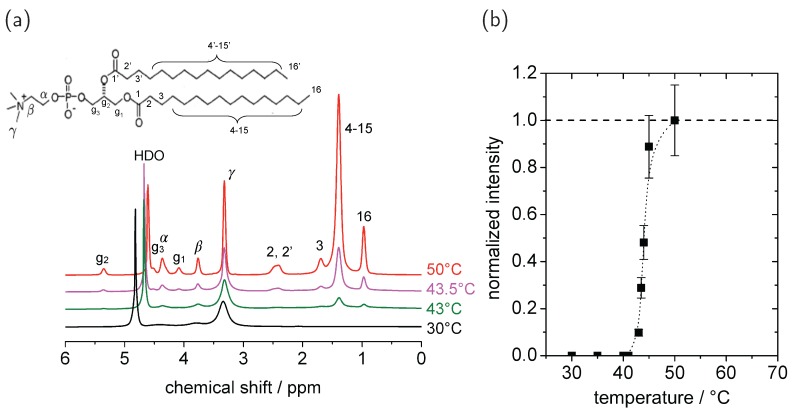
(**a**) ^1^H spectra of dipalmitoylphosphatidylcholine (DPPC) at different temperatures and (**b**) temperature dependence of the acyl chain peak intensity, normalized such that it reaches unity at high temperatures.

As an example, ^1^H spectra of DPPC at different temperatures are shown in Figure 3a. The peak assignment is well-known from literature [44,47]. Signals of the lipid acyl chain are found around 0–3 ppm and signals of the lipid glycerol backbone and the choline group appear in a range of 3–6 ppm. For DPPC in the gel phase (*T* = 30 °C), only some peaks of the mobile headgroup are visible. Due to the well-defined packing and comparably low mobility of the lipid acyl chains, the system is dominated by strong inter- and intra-molecular ^1^H–^1^H dipole–dipole couplings, which cause line broadening down to baseline level [44,45]. Therefore, the aliphatic and glycerol resonances are not visible at 5 kHz MAS, which is the recommended spinning frequency for this type of investigation (they would become at least partially visible at significantly faster spinning).

By heating above the main phase transition temperature of about 42 °C, the molecular dynamics change, i.e., fast uniaxial reorientation of the lipid molecules about their long axis sets in, and the peak intensities in the ^1^H spectrum increase to the maximum, so that the signals of all parts of the lipid are visible and well resolved [45,46]. Even though ^1^H–^1^H dipolar interactions still exist, they are sufficiently weak and rendered inhomogeneous by fast axial diffusion, leading to good resolution even at moderate MAS.

In Figure 3b, the temperature dependence of the normalized intensity of the acyl chain signal is shown. After baseline correction, the signal intensities are determined by integration over the respective centerband peak as well as over the spinning sidebands. Especially for temperatures below or near the phase transition, baseline correction is necessary to eliminate contributions from underlying broad signals of immobile protons. At high temperatures, when all molecules are sufficiently mobile, the peak intensities are proportional to the number of protons at the respective chemical site. The normalized relative peak intensities thus reflect the fraction of mobile lipid molecules in the sample.

At around the phase transition, the signal increases over a range of about 5 °C before it reaches its maximum. Such a transition temperature range instead of a sharp transition is caused by a temperature gradient over the sample in the rotor and by sample inhomogeneity. We define the inflection point of the sigmoidal trend as the phase transition temperature.

**Figure 4:**
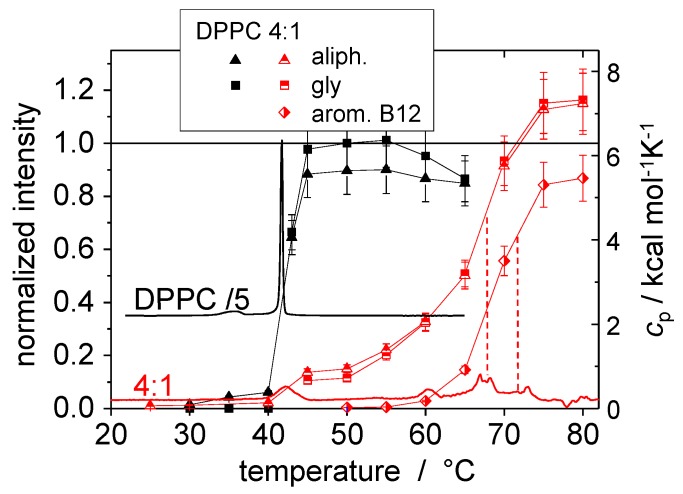
Temperature-dependent proton integrals (left scale) and differential scanning calorimetry (DSC) curves (right scale) for pure DPPC and the mixture DPPC/B12 4:1. The glycerol resonance measured for the pure DPPC sample was used as reference intensity for all peak integrals. Figure adapted with permission from ref. [22]. Copyright 2015 John Wiley& Sons.

^1^H spectra provided valuable information for the sample system of DPPC and the bolapolyphile B12, which is therefore reviewed here [22,23]. In Figure 4, results for the normalized ^1^H signal intensities of the aliphatic region, the lipid glycerol group, and the aromatic protons of B12 are shown for a pure DPPC sample and a 4:1 mixture of DPPC and B12, along with DSC curves.

Using DSC, three new phase transitions in a temperature range of 60–75 °C were found for DPPC/B12 in addition to the main phase transition of the pure lipid at about 42 °C. Thus, the conclusion was drawn that the sample phase separates into a pure lipid phase and B12-enriched phases for temperatures below 75 °C. Phase separation was also seen in fluorescence microscopy images showing star shaped domains in GUVs [22]. The ^1^H NMR spectra confirmed these observations and provided explanations on the molecular level.

The lipid glycerol and aliphatic signals of the DPPC/B12 mixture only increase to about 10% at the main phase transition temperature leading to the conclusion that only a small part of the lipid molecules becomes mobile. The other lipids are probably located in the B12-enriched phase where they are immobilized within the honeycomb cells formed by B12 (see Figure 2). Furthermore, the lipid signals increase again more rapidly at the temperature of the second new DSC peak, which can thus be related to the mobilization of the lipid molecules. The signal intensity of the aromatic protons of B12 is small and just slowly increases to its maximum in the temperature range of the third DSC peak. Therefore, we can conclude that this transition is associated with the mobilization of the aromatic cores of B12 which goes along with the dissolution of the supramolecular structure. Above all phase transitions at a temperature of *T* ≥ 75 °C, all peaks reached maximum intensity within the error margin of about 10 %, indicating a homogeneous mixture with fully mobilized molecules.

Similar experiments were performed for the mixture of DPPC and the bolapolyphile E12/7. Fluorescence microscopy images showed smooth GUVs without the typical corrugations related to a gel phase and without domains. ^1^H spectra revealed a slow gradual mobilization of the E12/7 aromatic cores above the main DPPC phase-transition temperature. It was thus concluded that E12/7 forms *π*-stacked, rigid domains, presumably filaments with width in the nanometer range, that randomly pervade the DPPC membranes [22,23].

### 3.2. Nuclear Overhauser Enhancement Spectroscopy

To understand intermolecular interactions, the localization of guest molecules in a lipid bilayer is an essential piece of information. For this purpose, the ^1^H–^1^H NOESY pulse sequence can be applied. It is well suited for the investigation of lipid samples due to the good resolution of ^1^H MAS NMR spectra and long spin-lattice relaxation times. Most commonly, the NOESY experiment is used in solution NMR to determine fixed intramolecular distances in organic chemical structures, for example, in proteins or DNA. However, it also yields information for membrane systems, where the interest lies in the temporary spatial proximity of nuclei in the lipids and the guest molecules.

**Figure 5:**
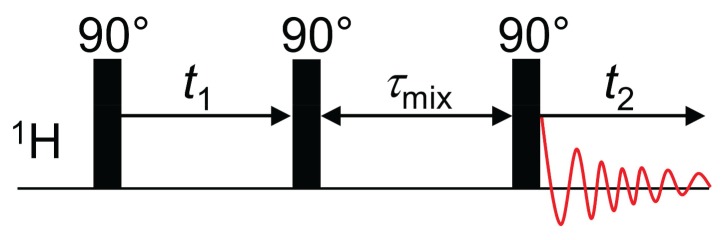
Schematic representation of the pulse sequence of the 2D Nuclear Overhauser SpectroscopY (NOESY) experiment.

Typically, a simple three-pulse sequence in phase-sensitive mode is used [48], see Figure 5. The first 90° pulse creates transverse magnetization, which evolves under the influence of the isotropic chemical shift during the waiting time $t_{1}$. In this way, each spin is labeled with its characteristic resonance frequency $\omega_{1}$. The subsequent 90° pulse flips the magnetization back into *z*-direction. Afterwards, magnetization exchange can take place during the mixing time $\tau_{\text{mix}}$. Finally, a last 90° pulse is applied and acquisition takes place during the time $t_{2}$, again providing evolution under the influence of the isotropic chemical shift. Fourier transformation yields a two-dimensional spectrum $S(\omega_{1},\omega_{2})$ which contains cross-peaks when magnetization exchange has occurred during the mixing time [48,49].

For lipid samples, cross-peaks are mainly caused by dipolar cross relaxation between two proton spins in close proximity (<5 Å). Chemical exchange does not occur and also spin diffusion in the lipid molecules has little influence on the experimental results [50,51]. From the cross-peak intensities, cross relaxation rates can be determined by using different methods, such as the full matrix approach, the spin-pair interaction model or the single mixing time approach [50]. Because of the high molecular mobility and disorder in lipid membranes, the cross relaxation rates provide a measure of the contact probability between the interacting spins [49].

The first NOESY MAS spectra of DMPC were recorded more than 20 years ago [45]. In several more recent studies, the distribution and orientation of various guest molecules in a membrane have been determined. For example, for the interaction of ethanol with a phospholipid membrane, it was found that ethanol is preferentially localized at the lipid–water interface, but can penetrate into the region of the upper chain segments as well [52,53]. The location distribution of small aromatic compounds such as flavonoids [54], indole [55], or multidrug transporter substrates [56], was determined. Also, peptides in interaction with model membranes were investigated by the NOESY experiment and precise structural information was obtained. For example, for a mixture of DMPC and the ras peptide, it was found that the side chains of the peptide insert into the hydrocarbon core of the membrane while the peptide backbone is localized at the interface [57].

**Figure 6:**
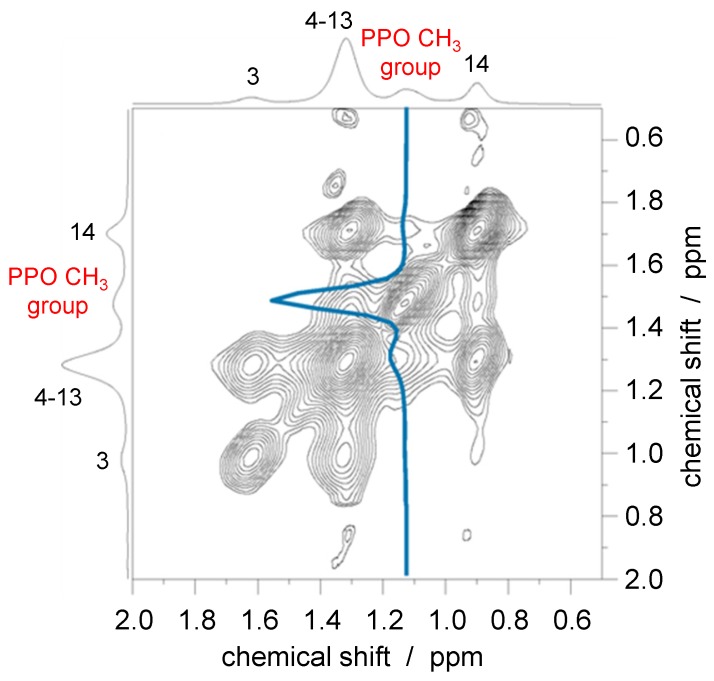
Acyl chain signal region of a 2D ^1^H–^1^H NOESY spectrum of the sample dimyristoylphosphatidylcholine (DMPC)/F-GP polymer (5 mol %) measured at 4 kHz magic-angle spinning (MAS), a temperature of 40 °C and a mixing time of 200 ms. The base level of the counter plots is set to 5% of the highest intensity. The blue line represent a slice at the position of the PPO-methyl resonance in the $\omega_{1}$-dimension. Figure adapted from ref. [16] published by the Royal Society of Chemistry.

In our research context, the NOESY experiment has recently been used to directly prove the incorporation of amphi- and triphilic block copolymers in a lipid bilayer [16]. As an example, we review the result for a mixture of DMPC and the pentablock copolymer F-GP. The region of the acyl chain signals of a 2D NOESY spectrum is shown in Figure 6. The measurements were performed for a sample in the liquid-crystalline phase at a temperature of 40 °C. In the ^1^H spectrum, many polymer and lipid signals overlap, especially in the region of the lipid headgroup resonances, which therefore cannot be further considered. However, the signal of the PPO methyl group shows a suitable isolated peak at about 1.1 ppm. Off-diagonal cross-peaks between overlapping resonances of the lipid acyl chain (including the methyl terminus) and the PPO methyl group can be seen clearly in Figure 6, confirming temporarily close molecular contacts between the hydrophobic groups of the lipid (acyl chains) and the hydrophobic block of the polymers (PPO). Therefore, it can be concluded that the polymer is not only adsorbed onto the surface but penetrates deeply into the lipid membrane. The relatively small intensity of the cross-peaks might be explained by the weak and rather transient contacts in the fluid bilayer. Furthermore, the polymers might form domains and only interact with the lipids at the domain boundaries [19].

### 3.3. ^*13*^C MAS Spectra

^13^C NMR using MAS and heteronuclear decoupling yields well-resolved spectra for lipid samples with the chemical shifts depending on molecular dynamics and packing. While a ^1^H spectrum has a chemical shift dispersion of only about 10 ppm, a ^13^C spectrum spans beyond 200 ppm. Multispin homonuclear dipolar couplings are not relevant for a dilute spin such as ^13^C, enabling a resolution comparable to solution NMR. However, the lower spin density and gyromagnetic ratio of ^13^C as compared to ^1^H also results in a reduced sensitivity. Therefore, more scans need to be accumulated, rendering the acquisition of a ^13^C spectrum more time consuming.

Direct polarization (DP) stands for the spin excitation by a single 90° hard pulse. For DP spectra recorded with a recycle delay long enough to allow for complete $T_{1}$ relaxation, the area under each resonance line is proportional to the number of nuclei in the respective chemical site. The width of the resonance lines depends on magnetic field homogeneity, the MAS rate, the efficiency of the ^1^H decoupling and the ^13^C transverse relaxation time.

**Figure 7:**
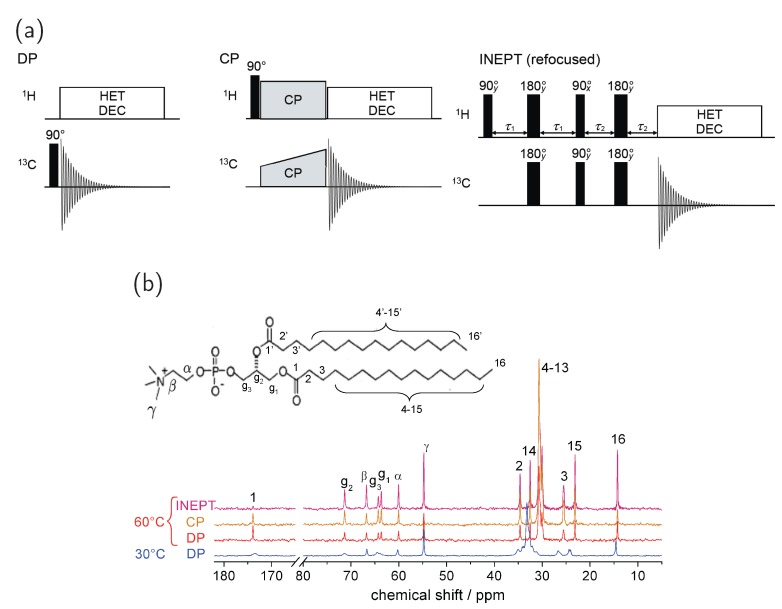
(**a**) Pulse sequences for the excitation of transverse ^13^C magnetization; (**b**) ^13^C MAS spectra of DPPC. Results for the temperatures 30 °C and 60 °C and for the excitation schemes DP, CP and (insensitive nuclei enhanced by polarization transfer) INEPT are compared. Note that due to broader lines, the spectrum at 30 °C is measured with a considerably higher number of scans.

Figure 7b shows DP spectra of DPPC for a temperature below and a temperature above the main phase transition. The peak assignment was done according to literature [44,47]. Due to lower molecular mobility in the gel phase, the peaks of the glycerol backbone and the acyl chains are broader in the spectrum acquired at 30 °C. However, the most remarkable difference between the two spectra can be seen for the peaks of the acyl chain region. The largest peak from the center-chain CH_2_ groups is shifted downfield by about 3 ppm in the gel phase vs. the liquid-crystalline phase. This effect mainly originates from the increased population of all-trans configurations of the hydrocarbon chain in the gel phase. In the liquid crystalline-phase however, the chains are disordered and change rapidly between trans- and gauche conformation. This influence of chain conformation on the chemical shift is called the γ-gauche effect [58].

As the sensitivity of ^13^C NMR is relatively low, several signal enhancement methods were developed. By inducing a ^1^H–^13^C polarization transfer, the high magnetization of the abundant lipid protons is exploited. The cross-polarization (CP) transfer is a technique frequently used in SSNMR. Because the polarization transfer is mediated by through-space dipolar interactions, it is most effective for rigid samples where the dipolar coupling is not averaged by molecular dynamics. After a ^1^H 90° pulse, the magnetization transfer occurs by simultaneously irradiating both ^1^H and ^13^C spins over a long time of about 100 μs up to 5 ms (see Figure 7a) with optimal results being obtained when the Hartmann–Hahn condition $\left|\gamma_{H}B_{1}\left({}^{1}H\right)\right|=\left|\gamma_{C}B_{1}\left({}^{13}C\right)\pm n\omega_{r}\right|$, with n=1,2,3… is fulfilled. Hereby, $B_{1}$ denotes the pulse amplitude and $\omega_{r}$ is the MAS rotation frequency. Additionally, a gradual increase of the proton or carbon spin-lock field in the rotating frame is applied. This ramped-amplitude (RAMP) CP reduces the signal sensitivity on pulse amplitude and $B_{1}$ field inhomogeneities and it ensures that also in complex systems the Hartmann–Hahn condition can be satisfied for all ^13^C spins [59,60].

For magnetization transfer in solution-state NMR, the refocused “insensitive nuclei enhanced by polarization transfer” (INEPT) method is applied [61]. This technique uses the through-bond J-coupling between nuclei to induce magnetization transfer and is therefore most effective for ^13^C atoms of mobile molecules in solution. However, INEPT was also applied successfully for lipid samples under MAS conditions, where the evolution delays just need to be rotor-synchronized [62]. The pulse sequence of the refocused INEPT method is shown in Figure 7a. For the delay times $\tau_{1}$ and $\tau_{2}$ averaged values, depending on the J-coupling are used, so that signals from CH, CH_2_ as well as CH_3_ groups can be seen.

CP and INEPT signal intensities contain qualitative information on molecular mobility, because the enhanced efficiency of the different methods depends on C–H bond reorientation dynamics which can be described by a correlation time $\tau_{c}$ and an order parameter *S* (see the next section for its definition). Nowacka et al. found out that under typical experimental conditions, there is nearly no INEPT signal and maximal CP signal for molecules performing slow and/or anisotropic motions with $\tau_{c}>10$ μs and/or S>0.5. In contrast, for molecules showing fast isotropic motions with $\tau_{c}<0.01$ μs and S<0.05, the CP signal is very small and the INEPT signal is maximal. When intermediate molecular motions with a $\tau_{c}$ in the order of microseconds are present, only a small polarization transfer can be reached with both methods [63].

With these considerations, the association of a guest molecule with the membrane can be investigated by simply comparing peak intensities in the CP and INEPT spectra. For example, a molecule that is incorporated into a bilayer is motionally constrained and will therefore give rise to larger CP and smaller INEPT signals than a molecule that moves isotropically in the surrounding water. The lipid bilayer itself represents a system in-between solid and liquid as it shows a fluid liquid-crystalline state above the main phase transition temperature. For such samples, the refocused INEPT sequence generally enhances the ^13^C signal more efficiently than the CP method [64]. The lipid molecules perform fast motions with $\tau_{c}<0.01$ μs and 0<S<0.3. In this regime, the ratio between CP and INEPT intensity can be used as a measure of the order parameter *S* [63].

As an example, for results of the different enhancement methods, Figure 7b shows a DP, a CP and a refocused INEPT spectrum of DPPC at 60 °C. The methyl groups at the chain ends and γ carbon in the headgroup are very mobile having a small order parameter. Accordingly, the INEPT spectrum shows a much higher signal for these peaks than the CP spectrum. On the contrary, the CP spectrum shows a stronger resonance for the large peak of the acyl chain middle region which is motionally restricted.

**Figure 8:**
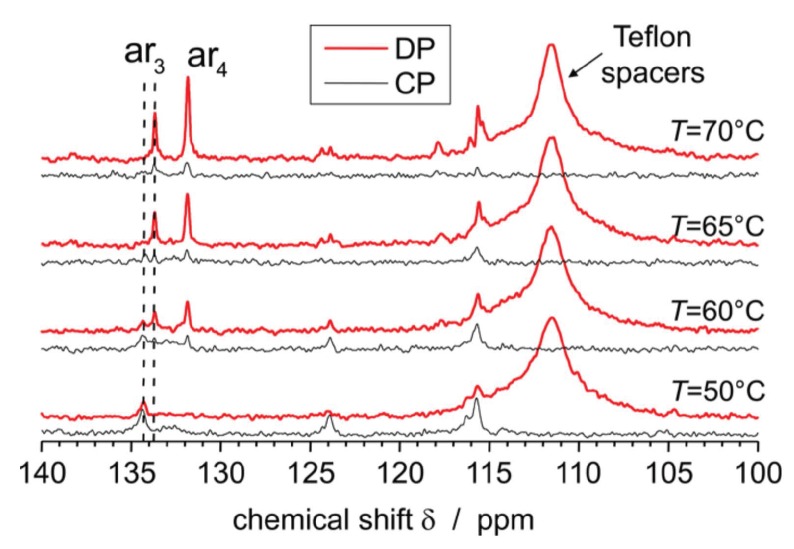
Aromatic signal region of ^13^C MAS spectra of a 4:1 mixture of DPPC and B12, comparing direct polarization (DP) (upper, red) and cross-polarization (CP) (lower, black) spectra taken at the indicated temperatures. The resonances ar_3_ and ar_4_ correspond to aromatic CH groups marked with green up-triangles in the B12 structure in Figure 2. Figure reproduced with permission from ref. [23]. Copyright 2016 Americal Chemical Society.

^13^C MAS spectra were used to study the packing and mobility of DPPC bilayers in interaction with the X-shaped B12 molecules [23]; in particular, they helped to clarify further the molecular underpinnings of the new DSC transitions discussed in the context of Figure 4. In Figure 8, the aromatic signal region of DP and CP spectra for different temperatures is shown for a 4:1 mixture. Remarkable is the temperature-dependent chemical shift of the signal ar_3_, which originates from an aromatic B12 ring. The peak at approximately 134.4 ppm slowly decreases in intensity with rising temperature and disappears completely at 70 °C, while the signal at approximately 133.7 ppm appears at about 60 °C and then increases further. It is assumed that the two signals arise from the same carbon for which the chemical surrounding changes with increasing temperature due to a change in π–π packing, which influences the related ring current effect and consequently the chemical shift. Furthermore, the mobility of the B12 molecules increases with rising temperature which can be deduced from the decreasing signal intensities in the CP spectrum and the sharpening of the peaks in the DP spectrum for temperatures above 50 °C. Thus, by help of the ^13^C MAS spectra, the additional phase transition of the mixture at about 60 °C found by DSC can be attributed to a change in the π-π packing as well as to the mobility of the B12 cores.

## 4. Dynamic Order Parameters in Lipid Membranes

### 4.1. C–H Order Parameters

**Figure 9:**
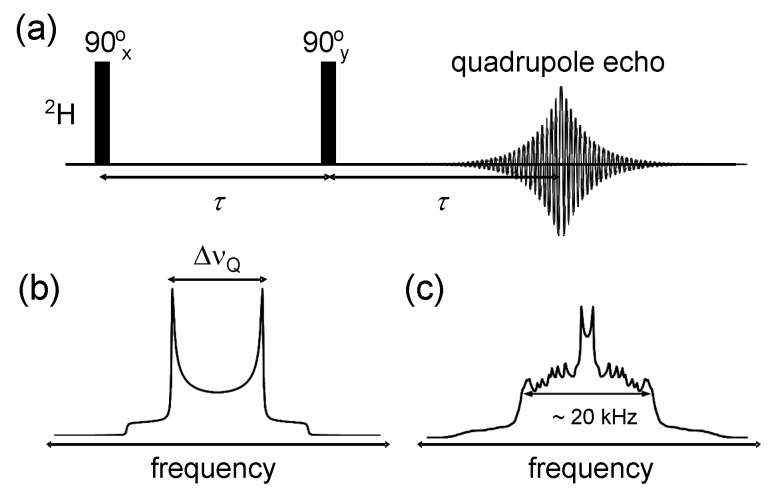
^2^H NMR determination of C–H bond dynamic order parameters. (**a**) Pulse sequence of the quadrupolar-echo experiment; (**b**,**c**) Illustrative simulated spectra of the quadrupole echo from a sample of multilamellar vesicles in the L_*α*_ phase with selectively single-site deuterated phospholipids (**b**) and per-deuterated phospholipids (**c**).

The NMR determination of the dynamic C–H bond order parameters from lipid bilayers has been conventionally done by ^2^H NMR, namely by measuring quadrupole echoes (also referred to as solid echoes) from multilamellar vesicles (MLVs) composed of deuterated phospholipids [24,65,66]. The reason to record an echo rather than just a free-induction decay after a 90° pulse is mainly technical, enabling a dead-time free detection and the recording of undistorted lineshapes. The Fourier transform of the quadrupolar echo, see Figure 9a, provides a spectrum that consists of a superposition of “Pake” (powder) patterns from the distinct deuterated sites. Each Pake pattern manifests two horns separated by

(4) $$\Delta \nu_{Q}=\frac{3}{4}\chi S_{\text{CH}},$$

where *χ* is the quadrupole coupling constant equal to 167 kHz [24] for C–^2^H moieties and $S_{\text{CH}}$ is a time-averaged dynamic order parameter defined by

(5) $$S_{\text{CH}}=\frac{3}{2}{\langle \cos^{2}\theta \rangle }_{t}-\frac{1}{2}={\langle P_{2}\left(\cos\theta \right)\rangle }_{t},$$

with *θ* being the angle between the C–H bond and the bilayer normal. Note that such a definition of *θ* can be applied strictly only for C–H bonds with fast uniaxially symmetric motion around the bilayer normal. $S_{\text{CH}}$ thus describes the reduction of the quadrupolar interaction by the fast uniaxial rotation combined with additional conformational fluctuations. The timescale of these motions needs to be significantly faster than the inverse of 3πχ/2 (≈1 μs), otherwise, strong signal loss during the quadrupolar echo and broad and featureless spectra are observed, indicative of “intermediate-regime” motions.

Determining how the distinct C–H bond order parameters vary in a phospholipid molecule upon insertion of a guest molecule enables the probing of interactions with atomistic detail. For instance, if cholesterol is inserted in a phosphatidylcholine (PC) bilayer, the magnitudes of the phospholipid acyl chain order parameter increase, while the head group order parameters remain essentially the same, clearly showing that cholesterol molecules locate next to the PC acyl chains and that the hydroxyl group does not interact strongly with the choline dipole [67].

Because the relevant time interval for the dynamic averaging implied in the definition of $S_{\text{CH}}$ is less than ∼1 μs, the order parameters can be interpreted by using all-atom and united-atom MD simulation trajectories [68]. For instance, MD simulations have been used to obtain mathematical relations that correlate dynamic order parameter profiles with bilayer acyl chain projected lengths and molecular areas [69].

The use of ^2^H NMR to unambiguously resolve C–H bond order parameters of phospholipids or other molecules residing in the lipid bilayer can only be realized by using selectively deuterated sites [24,65] as illustrated in Figure 9b. Selective deuteration approaches are however cumbersome [70], and in most ^2^H NMR studies fully- or per-deuterated phospholipid molecules are used instead, see Figure 9c for an example. The analysis then either involves a “de-paking” procedure subject to assumptions, or the extraction of the first and second spectral moments from which the averages $\langle |S_{\text{CH}}|\rangle$ and $\langle S_{\text{CH}}^{2}\rangle$, respectively, can be determined [71,72].

### 4.2. SLF NMR Spectroscopy

Although so far not used as frequently as the more conventional ^2^H NMR approach, other more advanced NMR techniques exist that enable the selective measurement of site-specific $\left|S_{\text{CH}}\right|$ values from samples with natural abundance of isotopes, namely the so-called ^1^H–^13^C separate local field (SLF) NMR experiments [73,74]. Equivalently to Equation (4), order parameter values are then obtained via $S_{\text{CH}}=D_{\text{res}}/D_{\text{CH}}$, where $D_{\text{res}}$ is the motion-averaged RDC (in the customary unit of rad/s, meaning that the corresponding spectral splitting equals $D_{\text{res}}/2\pi$). The typical SLF NMR result is a two-dimensional spectrum where the direct (detection) dimension consists of highly resolved ^13^C chemical shifts and the second (indirect) dimension with an associated evolution time $t_{1}$ (turning into $\nu_{1}$ after optional Fourier transform) contains information enabling the determination of the $\left|S_{\text{CH}}\right|\propto D_{\text{res}}$ values of each of the resolved carbons in the direct dimension. The resolution of different carbons by their different chemical shifts is achieved by using magic angle spinning and ^1^H decoupling during ^13^C acquisition.

The variety of ^1^H–^13^C SLF NMR methods published so far may be separated into two main groups, namely carbon-detected local field (CDLF) and proton-detected local field (PDLF), and are described in the next two sections. Note that, since the static-limit ^1^H–^13^C dipolar couplings (D/2π) are only of the order of 20 kHz, the conditions on the correlation time of motion to be below the μs range are somewhat relaxed, as the fast limit is already reached for motions that are about six times slower than for ^2^H NMR.

#### 4.2.1. Carbon-Detected Local Field NMR

**Figure 10:**
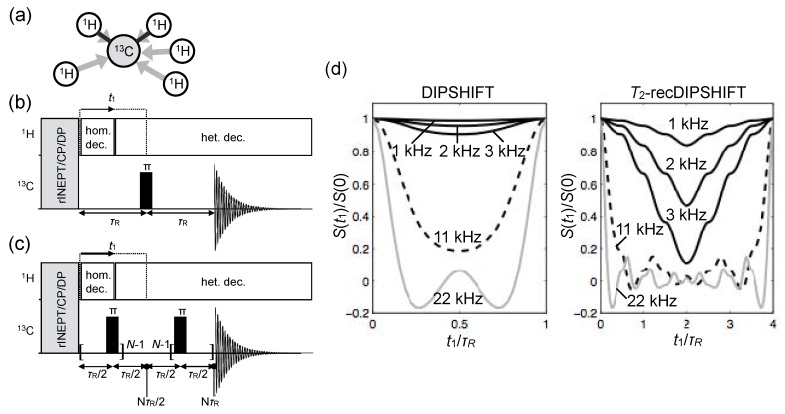
Carbon-detected local field spectroscopy. (**a**) Illustration of the basic carbon-detected local field (CDLF) configuration where carbon magnetization evolves under multiple proton dipolar couplings; Pulse sequences of the (**b**) dipolar chemical-shift correlation (DIPSHIFT) and (**c**) $T_{2}$-recDIPSHIFT methods; (**d**) Comparison of DIPSHIFT and $T_{2}$-recDIPSHIFT $t_{1}$ curves for a number of distinct C–H couplings. Note that the couplings shown correspond to the effective heteronuclear dipolar coupling under a given homonuclear decoupling sequence.

One possible way, and in fact the “classical” approach, of measuring C–H bond dynamic order parameters is by recording the dipolar field exerted by ^1^H spins on ^13^C nuclei [74,75]. In this case, the local field measured corresponds to the contributions of the dipolar fields induced by the individual spins in the proton network as illustrated in Figure 10a. However, since the dipolar field strength is inversely proportional to the cubic distance, for most cases the dominant contributions are from covalentely bound protons, and therefore isolated CH or CH_2_ spin groups can be assumed for interpretation of experimental data.

One popular CDLF NMR method is the dipolar chemical-shift correlation (DIPSHIFT) experiment [76,77] with the pulse sequence shown in Figure 10b. The dipolar field is probed during $t_{1}$ where the carbon transverse magnetization, $S(t_{1})$, evolves under the influence of the local heteronuclear dipolar field. DIPSHIFT is a so-called constant-time experiment, meaning that the $t_{1}$ time is increased at the expense of the following heteronuclear decoupling period, and ranges from 0 to one rotor period $\tau_{R}=1/\nu_{R}$. The functional form of $S(t_{1})$ can be described by analytical formulae based on Average-Hamiltonian theory; they enable the determination of the dipolar couplings from fitting the experimentally obtained $S(t_{1})$ curves [78,79,80]. For a CH group

(6) $$S\left(t_{1}\right)/S\left(0\right)={\langle \cos\phi \rangle }_{\beta ,\gamma }\;,$$

and for a CH_2_ group

(7) $$S\left(t_{1}\right)/S\left(0\right)={\langle \cos\phi_{1}\cos\phi_{2}\rangle }_{\alpha ,\beta ,\gamma }\;,$$

where the averages are over the Euler angles α,β and *γ* transforming the dipolar tensor components in the principal axis frame to the tensor components in the laboratory frame, and the phase angles depend on the C–H dipolar coupling strength and on the MAS frequency used. Explicit expressions for the $\phi_{i}\propto D_{\text{res}}$, which are phase factors related to the time integral of the dipolar tensor as modulated by the MAS, are given e.g., in refs. [78,80].

As illustrated in Figure 10d, although the curves $S(t_{1})$ from DIPSHIFT experiments can be very useful to determine high C–H dipolar couplings on the order of tens of kHz, for smaller couplings of a few kHz, the amplitude of the $S(t_{1})$ curve becomes too low for accurate determinations. In this case, it is convenient to amplify the $S(t_{1})$ curve by the introduction of additional 180° (*π*) pulses during the $t_{1}$ evolution leading dipolar recoupling [77,78,81]. The pulse sequence termed $T_{2}$-recDIPSHIFT is illustrated in Figure 10c.

**Figure 11:**
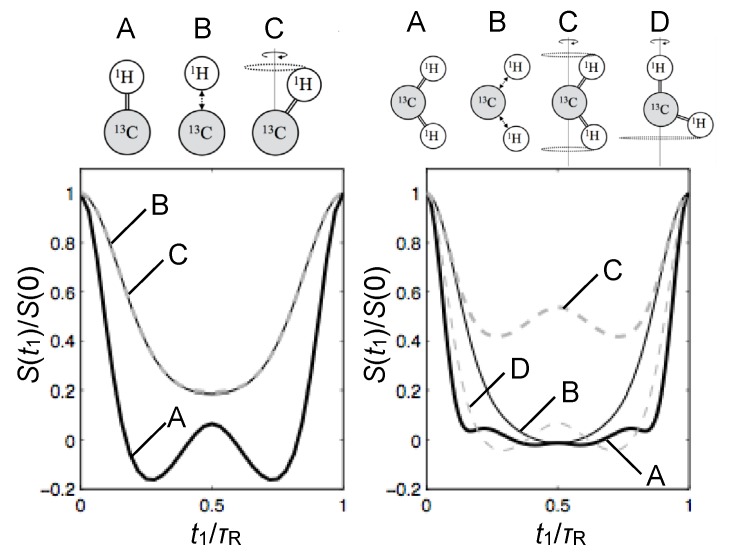
Effect of C–H motion on a DIPSHIFT curve for CH and CH_2_ groups. The effect is modeled by assuming uniaxially symmetric motion of the C–H bonds as indicated in the sketches and compared with the rigid cases A and B (left and right). For both CH and CH_2_, case B (no rotational motion) has the same dipolar coupling magnitude as case C (covalent bonds with rotational motion) by moving the proton away by the required distance.

The DIPSHIFT (and $T_{2}$-recDIPSHIFT) curves from CH_2_ groups are sensitive to the motional geometry of the H–C–H plane. In the case of lipid bilayers in the L_*α*_ phase, all C–H bonds undergo motions with uniaxial symmetry around the bilayer normal vector. This does not mean, however, that the C–H bonds of a given CH_2_ group need to have the same order parameter. In fact, the equivalence or not of the two C–H order parameters of a methylene group is fully related to its motional geometry, contrarily to what has been described recently [82]. This is illustrated in Figure 11, where it is also shown how the inequivalence of the order parameters in a CH_2_ group affects the shape of its DIPSHIFT curve.

**Figure 12:**
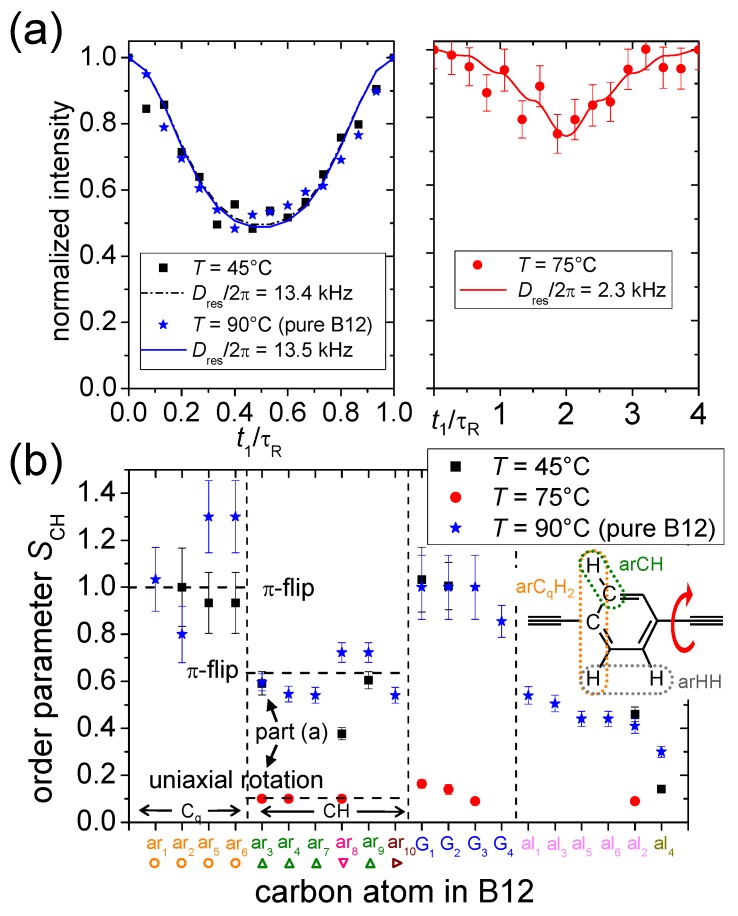
(**a**) ^13^C–^1^H DIPSHIFT (**left**) and $T_{2}$-recDIPSHIFT (**right**) dephasing curves for aromatic resonance ar_3_ of B12 in a 1:4 mixture with DPPC; (**b**) $S_{\text{CH}}$ values for all resolved B12 resonances as well as expected values for specific motional models for B12 (dashed lines). The inset shows schematically the different dipolar tensors probed. See Figure 2 for resonance assignments of the B12 aromatic core. Figure reproduced with permission from ref. [23]. Copyright 2016 Americal Chemical Society.

Following these principles, order parameters have been measured with DIPSHIFT to study peptide structure and orientation in lipid bilayers [83,84,85]. The use of both the DIPSHIFT and also $T_{2}$-recDIPSHIFT methods shall here be illustrated by referring to our recent studies of dynamic order parameters of T-shaped [21] and X-shaped [23] bolapolyphiles inserted in lipid bilayers. As for the lipid resonances, it is noted that there was in all cases no detectable variation in $S_{\text{CH}}$ values, indicating that the membrane parts remained unchanged upon bolapolyphile addition, at least on the faster timescale probed by $S_{\text{CH}}$. The T-shaped molecule A6/6 was found to not be immersed in the bilayers (the detectable carbons having no detectable $T_{2}$-recDIPSHIFT dephasing), while it was demonstrated that the X-shaped bolapolyphiles are, to the most part, significantly constrained, with the aromatic rings only performing *π* flip motions in the filamentous supramolecular structure discussed above. This type of motion is also observed in bulk crystals of B12, as demonstrated by the data in Figure 12a.

Upon dissolution of the supramolecular structure into the single-phase system, the observed $S_{\text{CH}}$ is reduced significantly, in agreement with free rotation and an orientation along the bilayer normal. The order parameter profile shown in Figure 12b for all resolved resonances confirms this picture. Notably, the quaternary ^13^C atoms in the rings exhibit a spin configuration equivalent to a CH_2_ group, providing an independent tensorial probe of the observed motions. The determination of $S_{\text{HH}}$ values, representing yet another independent probe, is discussed in a separate section.

Although DIPSHIFT experiments require fitting experimental data with an analytical model (or by using simulations) to determine the C–H dipolar couplings, when amplifying the coupling, as in $T_{2}$-recDIPSHIFT, it becomes also possible to observe couplings by performing a Fourier transform along the indirect dimension provided the recoupling is done over a sufficient long time. Then, as result of the Fourier transformation, one obtains spectra with dipolar splittings which are directly proportional to the coupling being measured, the proportionality factor being specific of the recoupling pulse sequence used. For instance, Zhao et al. [86] have suggested a CDLF method with an amplification method over $t_{1}$ that performs ^13^C –^1^H dipolar recoupling and ^1^H homonuclear decoupling simultaneously.

The described DIPSHIFT-based methods are rather easy to set-up and robust with respect to pulse imperfections. However, they suffer from a practical disadvantage for cases where CH_2_ groups have two distinct C–H dynamical order parameters and for crowded spectral regions where the resolution is lost. One very powerful method to overcome this drawback is the so-called proton detected local field NMR spectroscopy.

#### 4.2.2. Proton-Detected Local Field NMR

**Figure 13:**
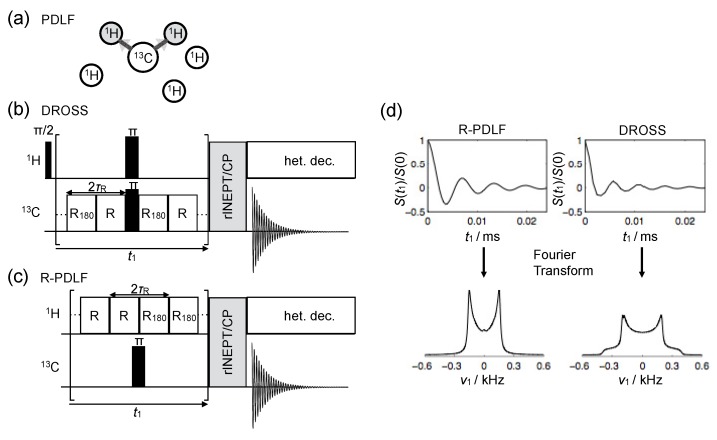
Proton-detected local field NMR. (**a**) Basic mechanism where the heteronuclear dipolar coupling is recoupled, modulating the proton magnetization which is then transferred to carbon; (**b**) The Dipolar Recoupling On-axis with Scaling and Shape preservation (DROSS) pulse sequence and (**c**) the R-PDLF pulse sequence; (**d**) Simulations of corresponding results using the SIMPSON software [89] for a heteronuclear dipolar coupling of 1 kHz.

In contrast to the previously described CDLF method, for a proton-detected local field method, rather than observing the local dipolar field in the carbon atom, one observes the individual dipolar fields induced by a carbon nucleus on its neighbouring protons [87], see Figure 13a. This is done by performing a magnetization transfer from protons to carbon only after the $t_{1}$ evolution as illustrated in Figure 13b,c. The signal evolution recorded during $t_{1}$ can be therefore described as a sum of signals.

(8) $$S\left(t_{1}\right)=\sum_{i}^{N}a_{i}S_{i}\left(t_{1}\right)\;,$$

where $S_{i}\left(t_{1}\right)$ represents the modulation of the magnetization of the *i*th proton due to its dipolar coupling with the close-by carbon, and $a_{i}$ represents the fraction of magnetization transfered to carbon by that proton. To ensure that the signal of a given carbon becomes simply a superposition of dipolar curves from its covalently bound protons, one can use a refocused INEPT sequence (with or without homonuclear decoupling) or a CP transfer with a short contact time [88].

Distinct PDLF methods have been designed and applied to lipid MLVs [90,91,92,93,94]. Two of these methods are shown in Figure 13b,c. The first is called Dipolar Recoupling On-axis with Scaling and Shape preservation (DROSS) [92], and it has been applied to study the effect of cholesterol [95] and of oxidized lipids [96] on lipid bilayers. One important feature of DROSS is that it can be modified in order to be sign-sensitive enabling to detect the sign of $S_{\text{CH}}$, which can reveal differences between motional geometries [92,96]. The second, called R-type PDLF or R-PDLF, makes use of simultaneous homonuclear decoupling and heteronucler recoupling (by so-called R-type sequences) and provides therefore better resolution than the aforementioned DROSS sequence [93]. Note that the R-PDLF lineshape of a C–H spin pair is not a Pake-pattern but resembles instead the *n* = 2 rotary-resonance lineshape [93], see Figure 13d.

**Figure 14:**
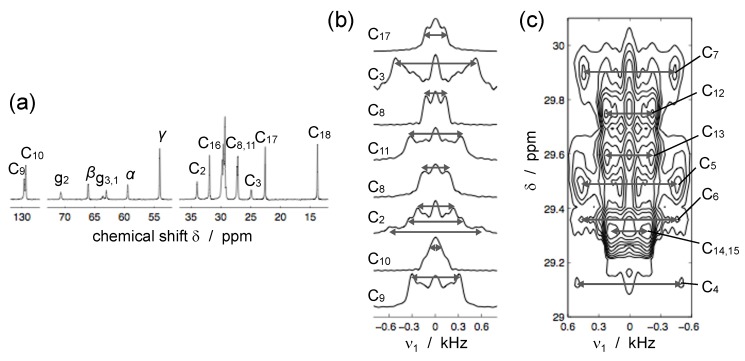
R-PDLF dipolar profile of DOPC MLVs at 37 °C. (**a**) ^1^H–^13^C refocused INEPT spectrum with labels for the distinct dioleoylphosphatidylcholine (DOPC) carbons; (**b**) Dipolar slices of the R-PDLF spectrum at the ^13^C chemical shifts of the indicated carbons showing distinct dipolar splittings; (**c**) Contour plot of the crowded spectral region with highly resolved splittings. The assignment was based on a previous determination done for POPC MLVs [67].

The important advantage of PDLF over CDLF techniques is that they enable the recording of highly resolved dipolar couplings both from CH groups as well as from CH_2_ groups that have two non-equivalent CH dynamic order parameters. Moreover, such methods enable the resolution of a very large set of CH dynamic order parameters from crowded spectral regions. This is exemplified in Figure 14 for a sample of DOPC MLVs using the R-PDLF method.

### 4.3. Intermediate Motional Regime

As mentioned, the condition to measure a well-defined $S_{\text{CH}}$ value is that the effective correlation time of rotational motion of the respective C–H moiety is significantly faster than about 1 μs. This simply makes sure that no “intermediate” motion takes place during the timescale of the actual NMR pulse sequence. If this condition is not fulfilled, transverse relaxation ($T_{2}$) effects start to dominate the results. In the case of ^2^H NMR, this leads to significant signal loss during the quadupolar echo sequence and line broadening, accompanied by complex spectral shapes, the detailed study of which has become popular and relevant for the case of synthetic labeled polymers [97]. In lipids, lineshapes indicative of large-angle intermediate motions have been reported for gel phases [24]. In liquid-crystalline phases, fast uniaxial rotation prevails, and relevant intermediate motions comprise smaller-amplitude fluctuations of the lipid long axis. In this case, the spectra remain reasonably well-resolved (see Figure 9), and the study of orientation-dependent $T_{2}$ can be informative [36]. The related phenomena will be discussed in some detail in the last section dealing with ^31^P NMR.

For the case of DIPSHIFT experiments, significant work of our group was devoted to studying the actual timescale of intermediate motions [78,98,99]. In DIPSHIFT dephasing curves (see Figure 12a for actual data), motions entering the correlation time that range above about 1 μs lead to a significant decay of the signal detected towards the end of the $t_{1}$ evolution time window. The main phenomenon is that the $T_{2}$ relaxation time, for which small values correspond to large signal loss, goes through a minimum when the correlation time of motion is of the order of the inverse *D* or the inverse spinning frequency. Motions slower than that have a progressively weaker effect ($T_{2}$ attains again large values), reaching the rigid-limit result for an effectively immobile C–H moiety. A comparison of experimental DIPSHIFT data exhibiting such a $T_{2}$ decay with simulations [98,99], or a more practical, recently developed analytical fitting approach [99], enables the estimation of correlation times of motion. This important option has yet to find applications in the field of lipids, but it should be noted that quantitative results can only be expected for the case of large-amplitude motions.

### 4.4. H–H Order Parameters

As shown in the previous section, ^1^H–^13^C dipolar couplings determined from NMR experiments can often be used to probe local order, structure and dynamics in lipid bilayer systems. However, the methods presented also show limitations, such as a low signal-to-noise ratio associated with the carbon detection and a significant inaccuracy in determining small coupling constants of 1 kHz or below, which is especially disadvantageous for the investigation of highly mobile guest molecules of low concentration in the bilayer. As an alternative, we will therefore introduce in this section double-quantum (DQ) NMR, which is used for measuring residual ^1^H–^1^H dipolar couplings and thus the related order parameter $S_{\text{HH}}$.

The proton-detected DQ experiment provides reasonably good sensitivity due to the high natural abundance and gyromagnetic ratio of the protons and resolution provided by MAS. Therefore, also signals of a small minority guest component within the membrane can be studied. Furthermore, rather small ^1^H–^1^H couplings down to about 100 Hz, characteristic of highly mobile systems, are readily accessible. Additionally, information on the distribution of the dipolar couplings can be obtained.

#### 4.4.1. Experimental Considerations

For all lipid sample systems, we used the BaBa-xy16 pulse sequence which is a two-dimensional DQ experiment performed under MAS, yielding chemical resolution in the direct dimension and information on the recoupled homonuclear dipolar coupling in the indirect dimension. Originally, the BaBa (“back-to-back”, referring to pulse placement) experiment was developed by Feike et al. [100]. Later on, Saalwächter et al. added the xy-16 phase cycle known from Carr-Purcell-Meiboom-Gill (CPMG) experiments [101] to achieve a truly broadband BaBa variant, termed BaBa-xy16 [102]. The main principles of the experiment to be outlined below have been established for static ^1^H NMR [103], but are straightforwardly extended to MAS [104].

**Figure 15:**
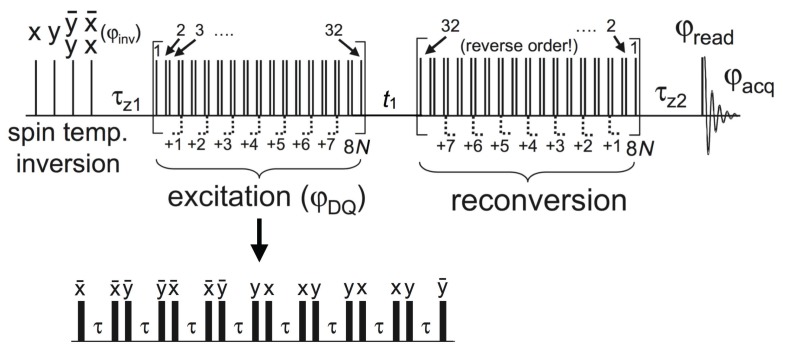
BaBa-xy16 pulse sequence as introduced in ref. [102]. All black bars symbolize 90° pulses. The pulse phases $\varphi_{i}$ are varied as described in the text. Figure reproduced with permission from [102]. Copyright 2011 Elsevier.

The BaBa-xy16 pulse sequence is shown in Figure 15. Briefly, it consists of an excitation sequence creating DQ coherences, an optional $t_{1}$ evolution time, and a (mainly just inverted) reconversion sequence which produces measurable magnetization. The excitation and the reconversion sequences contain a variable number of 90° pulses and have an equal length $\tau_{\text{DQ}}$, which is an integer multiple of the rotation period $\tau_{R}$. At the beginning, we have a preparation step, and in the end a read-out pulse before signal acquisition starts during $t_{2}$ [102].

There are essentially two ways to obtain dipolar-coupling information from this experiment: (i) DQ build-up curves are recorded by incrementing $\tau_{\text{DQ}}$ of the excitation and the reconversion blocks while setting the delay time $t_{1}$ in-between the two blocks to zero; (ii) DQ sideband patterns [104,105] are obtained by fixing $\tau_{\text{DQ}}$ at a suitable value and incrementing $t_{1}$ in small steps $\Delta t\ll \tau_{R}$, followed by Fourier transformation over this indirect spectral dimension [102]. In both cases, a 4-step DQ selection phase cycle, rotating the excitation (or reconversion) base phase in 90° steps, is applied. Alternation of the receiver phase between ±180° yields the DQ signal $I_{\text{DQ}}=\langle \sin\phi \left(0\right)\sin\phi \left(t_{1}\right)\rangle$. The brackets denote the powder average and $\phi (t_{1})$ is the dipolar phase factor, $t_{1}$ denoting the starting time of the block, which is relevant when $t_{1}\neq n\tau_{R}$.

#### 4.4.2. DQ Build-Up Curve Analysis

Following option (i) from above, we first focus on build-up curve analysis, for which only a single phase factor $\phi \left(0\right)=\phi \left(t_{1}\;\;\;\;\;=\;\;\;\;\;0\right)=\phi$ is relevant. In this case, intensity normalization is inevitable to obtain meaningful results [103,104]. This is achieved by taking a second signal function without receiver phase alternation, termed the reference signal $I_{\text{ref}}=\langle \cos^{2}\phi \rangle$. It contains a signal that has not evolved into DQ coherences, being dominated by modulated longitudinal magnetization. The actual signal function used for normalization is the sum $I_{\Sigma \text{MQ}}=I_{\text{DQ}}+I_{\text{ref}}$, which is a type of multi-spin dipolar echo function. Based upon this, we can calculate the point-by-point normalized DQ build-up curve:

(9) $$I_{\text{nDQ}}=\frac{I_{\text{DQ}}}{I_{\Sigma \text{MQ}}-I_{\text{tail}}},$$

which is corrected for motion-related relaxation, pulse sequence imperfections and higher-order dephasing terms. In the denominator, we subtract the contribution $I_{\text{tail}}$ of uncoupled spins showing a slowly relaxing, often exponential tail in $I_{\text{ref}}$. These may be related to isotropically mobile defects in an inhomogeneous system, see below for an example. $I_{\text{nDQ}}$ is theoretically expected to approach a value 0.5 in the long-time limit, which represents a test criterion for successful normalization.

To determine the dipolar coupling constant, different fit functions describing the build-up curve can be used. The simplest approach is the second-moment approximation

(10) $$I_{\text{nDQ}}=\langle \sin^{2}\phi \rangle \approx \frac{1}{2}\left\{1-e^{-2\langle \phi^{2}\rangle }\right\}$$

with the mean-square phase factor [102]

(11) $$\langle \phi^{2}\rangle =\frac{6}{5\pi^{2}}D_{\text{res}}^{2}\tau_{\text{DQ}}^{2}\;.$$

It fits the data well up to an intensity of $I_{\text{nDQ}}\approx 0.45$ [106]. An even better approximation of the experimental data is provided by the “Abragam-like” build-up function

(12) $$\begin{array}{ccc} I_{\text{nDQ}}\left(\tau_{\text{DQ}},D_{\text{res}}\right) & = & 0.5\left(1-\exp\{-{\left(0.295D_{\text{res}}\tau_{\text{DQ}}\right)}^{1.5}\}\right.\times \left.\cos(0.455D_{\text{res}}\tau_{\text{DQ}})\right)\;, \end{array}$$

which was found empirically by Chassé et al. [106]. Due to inhomogeneities, most samples feature a distribution of coupling constants. The simplest way to take this into account is by introducing a Gaussian distribution function. For the second-moment approach, the resulting fit function can be calculated analytically. An “Abragam-like” build-up function combined with a coupling constant distribution (resulting in a mere two-parameter fit) can be implemented numerically with a finite-step integration extending over about $D_{\text{res}}=\pm 3\sigma$, *σ* being the standard deviation.

The utility of the DQ experiment in investigating membrane systems should now be demonstrated on the example of mixtures of the lipid DMPC and the triblock copolymers GP and F-GP, which is an unpublished follow-up of our previous work [16]. In this case, it is particularly favorable to record DQ build-up curves, as the polymers show a high mobility and the ^1^H–^13^C couplings are too small to be resolved by the PDLF or DIPSHIFT experiments (data not shown).

**Figure 16:**
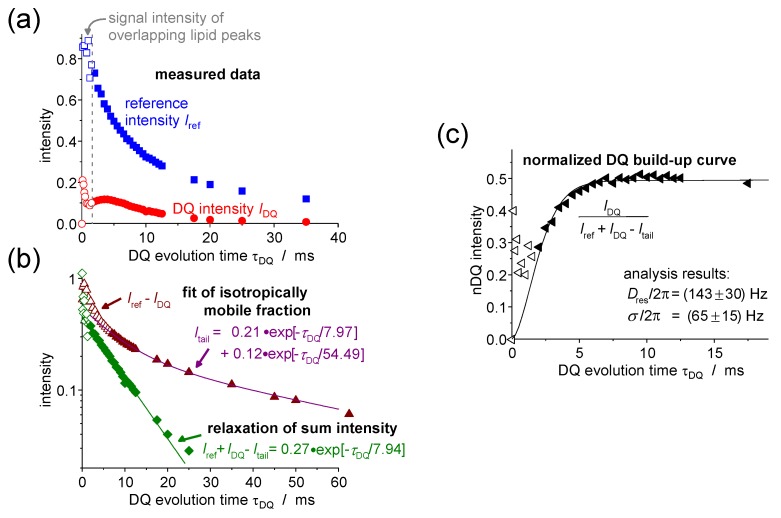
Results from a DQ experiment on DMPC/GP 60:1 at 40 °C. (**a**) Measured DQ and reference intensities; (**b**) tail fraction determined from the difference intensity to be the sum of two exponentials, and single-exponential fit of the sum intensity; (**c**) normalized DQ build-up curve and results from a fit based upon Equation (12).

Actual data and the analysis steps for the peak of the polymer PPO methyl group in the mixture DMPC/GP 60:1 are shown in Figure 16. In part (a), on the very left we can see that for small $\tau_{\text{DQ}}$ of less than 2 ms, the DQ intensity first increases very strongly and then decreases again. This is due to an overlap of the polymer peak with the foot of the large signal of the motionally more restricted lipid acyl chain (see the spectrum at the top of Figure 6), causing the large initial peak in the DQ build-up curve. However, the lipid signal relaxes fast, so that for $\tau_{\text{DQ}}$ exceeding 2 ms, the polymer peak can be separated clearly and the intensity is not influenced by the lipid molecule anymore, but shows a slow increase of the DQ intensity which is characteristic of the mobile polymer. For all data analyses, one can therefore just neglect the intensities measured during the first 2 ms.

The contribution $I_{\text{tail}}$ of uncoupled spins was in the given case determined by a bi-exponential fit to the intensity difference $I_{\text{ref}}-I_{\text{DQ}}$ as shown in Figure 16b. This is genereally the best way to identify signal contributions arising from isotropically mobile moieties. Here, they come from PPO methyl groups within chains that are not incorporated in the membrane but instead form small micelles with a radius of only a few tens of nanometers [15]. These are very mobile in water. Also, polymers that fold into a hairpin upon membrane incorporation could exhibit isotropically mobile parts of the PPO block in the middle of the bilayer. The formation of polymer domains within the membrane is a third possibility [19]. At the lipid/polymer border, the polymer dynamics is anisotropic, while there could be much less motional restriction for polymers within the domain.

The signal fraction $f_{a}$ of the anisotropically mobile PPO methyl groups was determined by back extrapolation based upon a single-exponential fit to the difference of sum and tail intensities, as also shown in Figure 16c. This fit is needed because the intensity $I_{\Sigma \text{MQ}}\left(\tau_{\text{DQ}}\;\;\;\;=\;\;\;\;0\right)$ is not known due to the overlapping lipid signal. As a result, we obtained an $f_{a}$ of about 0.45 for DMPC/GP 60:1 as well as for DMPC/FGP 60:1, which leads to the conclusion that at least about half of the PPO blocks are incorporated into the lipid bilayer where they perform restricted anisotropic motions.

In Figure 16c, the normalized build-up curve and the fit on the basis of Equation (12) including a coupling distribution is shown. A coupling constant of $D_{\text{res}}/2\pi =143$ Hz and a distribution width of σ/2π=65 Hz were determined. Within the uncertainty limits the same results were obtained for the sample DMPC/FGP 60:1. For both polymers, the PPO block seems to behave in a similar way and is possibly not influenced by additional anchoring of the fluorinated block of FGP.

For calculating and analyzing the order parameter of the *polymer backbone* (rather than a specific H–H internuclear vector) $S_{\text{HH}}=D_{\text{res}}/D_{\text{ref}}$ from $D_{\text{res}}$ measured for the methyl groups of the PPO block, one can use a reference coupling of about $D_{\text{ref}}=6.3$ kHz as estimated for polymers with similar local spin configuration [104,107]. The resulting order parameter of $S_{\text{HH}}=0.023$ is rather small. For a freely fluctuating Gaussian polymer chain of contour length $R_{\text{max}}$ subtended between two fixed points at a distance *R*, classical arguments [108,109] yield $S=\frac{3}{5}\frac{R^{2}}{R_{\text{max}}^{2}}$. Assuming that the polymer has a trans-bilayer conformation, we can take *R* to be the hydrophobic membrane thickness of 2.54 nm [110]. Taking $R_{\text{max}}=12.2$ nm for the fully stretched PPO block [19], the obtained order parameter of S=0.026 is indeed very close to the experimental value.

A critical discussion of this result is beyond the scope of the present review. Currently, we are applying this method in a systematic investigation of different Pluronics incorporated into various model membranes. In this way, we seek to establish a quantitative understanding of polymer incorporation into membranes and the associated anisotropic segmental fluctuations. The potential role of the nematic director field exerted by the acyl chains will have to be considered carefully.

#### 4.4.3. DQ Sideband-Pattern Analysis

DQ build-up curve analysis is only possible for small coupling constants up to about 1 kHz. Stronger dipolar couplings lead to a very fast increase of the DQ build-up curve which shows in this case only a few data points in the meaningful initial build-up region. This is due to $\tau_{\text{DQ}}$ being an integer multiple of the rotation period $\tau_{R}$, the minimum value of which is determined by the fastest feasible MAS frequency. Therefore, spinning side-band analysis using an incremented $t_{1}$ at fixed $\tau_{\text{DQ}}$ is preferred for the accurate determination of stronger dipolar couplings.

**Figure 17:**
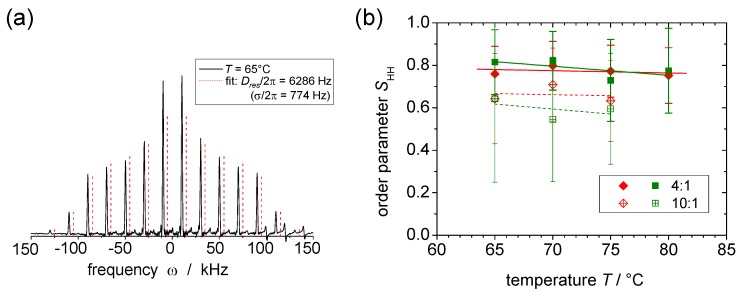
(**a**) DQ spinning sideband pattern for B12 in 1:4 mixture with DPPC at 65 °C (10 kHz MAS, 4 $\tau_{R}$ recoupling), including a fit to obtain the ^1^H–^1^H dipolar coupling and its distribution width; (**b**) Corresponding order parameters $S_{\text{HH}}$ for 4:1 and 10:1 DPPC/B12 mixtures evaluated for two aromatic signals each as a function of temperature. The error bars indicate the dipolar distribution width *σ* from the fit; the actual experimental error is smaller and apparent from the deviations from the trend lines. Figure adapted with permission from ref. [23]. Copyright 2016 Americal Chemical Society.

The 2D DQ–SQ correlation spectrum resulting from the Fourier transformation over the incremented $t_{1}$ shows a spinning sideband pattern in the indirect (DQ) spectral dimension. See Figure 17a for an example. It is only due to the signal of dipolar coupled protons and directly reflects the coupling constant [104,105]. Again assuming a Gaussian distribution, $D_{\text{res}}$ and the distribution width *σ* can be determined from the sideband pattern on the basis of a fit relying on a numerical calculation [104]. The distribution might arise from actual inhomogeneities in the sample, but also from couplings to remote protons or from a bias in the assumed isotropic powder average caused by an anisotropic transversal relaxation [22,104].

Our investigations on lipid bilayers in interaction with the X-shaped bolapolyphile B12 represent an example that demonstrates the relevance of the DQ sideband measurements [22,23]. In the bilayer, the B12 molecule adopts a mainly transmembrane orientation and performs additional wobbling motions, which can be investigated in detail by measuring $S_{\text{HH}}$ associated with the protons in the *para*-substituted phenyl rings. Their internuclear vector points along the long axis of the molecule and is thus not affected by fast uniaxial rotation but only by the additional fluctuations of the molecular long axis.

The data for $S_{\text{HH}}$ shown in Figure 17b cover a temperature range of 65–80 °C, which is above the DPPC phase transition temperature, which means that at least a fraction of the B12 molecules diffuses freely and performs fast uniaxial rotations. It thus shows enough mobility to be detected in the proton spectrum [23]. At temperatures below 70 °C the samples are characterized by phase separation, and at high temperatures by a uniform molecular distribution.

The results cover mixing ratios DPPC/B12 of 4:1 and 10:1. As a main outcome, it was found that the order parameter of B12 is lower in the 10:1 mixture than in the 4:1 mixture, suggesting smaller-amplitude motions of B12 when its concentration in the membrane is higher, probably due to crowding effects. Depending on the assumed motional model (rotation on or within a cone), the mean tilt angle of the molecules with respect to the membrane normal can be estimated to be about 20°–30° and 35°–45° for the 1:4 and 1:10 mixtures, respectively. The slight decrease of $S_{\text{HH}}$ with increasing temperature indicates a small increase of the motional amplitude of the wobbling motion.

In an analogous measurement series, we found very similar results for the mixture DOPC/B12. In this case, fluorescence microscopy results showed homogeneous GUVs, suggesting a uniform distribution of B12 over the bilayer at all temperatures [23]. Over the whole accessible temperature range, DOPC is in the mobile liquid-crystalline phase, and B12 diffuses freely and performs uniaxial rotation. The similarity of the results for the two different lipid membranes suggest that the interaction between lipid- and B12-molecules is rather weak.

## 5. Static ^31^P NMR as a Probe of Headgroup Mobility

**Figure 18:**
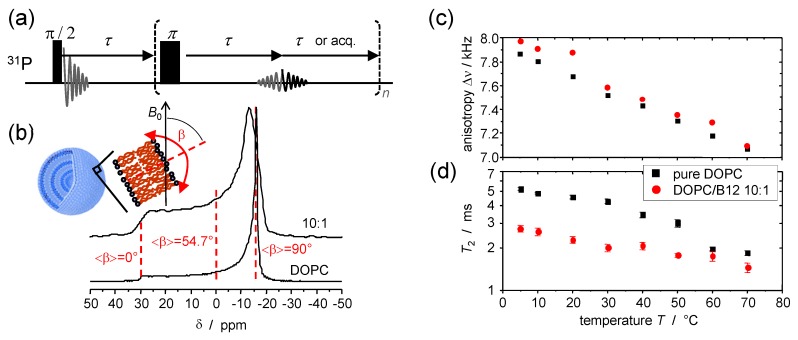
^31^P lineshape and relaxation studies. (**a**) Schematic pulse sequence for Hahn-echo and Carr-Purcell-Meiboom-Gill (CPMG) experiments; (**b**) Static ^31^P spectra of MLVs of DOPC, pure and as a 10:1 mixture with B12 taken at 30 °C. The red dashed line indicates the spectral position corresponding the the magic-angle orientation (〈β〉 = 54.7°) of the membrane normal with respect to $B_{0}$; (**c**) Temperature-dependent width of the spectra $\Delta \nu =\frac{3}{2}\delta \omega_{0}/2\pi$, see Equation (2); (**d**) Temperature-dependent $T_{2}$ relaxation times from Hahn-echo experiments evaluated for 〈β〉≈ 55°. All ^31^P experiments have to be, and have been, conducted with low-power ^1^H decoupling.

As mentioned at the outset, fast uniaxial rotation of the lipids in the liquid-crystalline phase leads to spectra reflecting an axially symmetric interaction tensor in static ^2^H and ^31^P NMR experiments conducted for single-site labeled acyl chains and the headgroup, respectively. For examples see Figure 9c and Figure 18b, respectively. The relevant spin interactions are the ^2^H quadrupolar coupling and the ^31^P CSA, respectively. The latter is described by Equation (2), noting again that due to the fast rotation around the lipid long axes, 〈δ〉 is a reduced, time-averaged anisotropy, and *β* is the orientation of the membrane normal with respect to $B_{0}$. Additional time averaging due to fast lamellar undulations may also be relevant. For both ^2^H and ^31^P NMR, orientation fluctuations on the intermediate timescale lead to signal loss during the relevant quadrupolar or Hahn-echo pulse sequences [36,111,112,113], respectively, associated with short $T_{2}$ relaxation times, and the related broadening of the individual resonance lines associated with different orientations *β*.

This implies that $\beta \left(t\right)=\langle \beta \rangle +\delta_{\beta }\left(t\right)$ is a function of time, arising from either lateral diffusion of the lipid along a curved membrane, or from collective fluctuations (undulations) of the membrane itself. Theoretical analyses suggest that the latter dominate in well-hydrated membrane systems at sufficiently high temperature [36], which means that, ideally, information on viscoelastic properties such as the membrane modulus or the viscosity can be estimated from the data [36,111,112,113]. Considering the high natural abundance and the comparably high gyromagnetic ratio of ^31^P, it is not surprising that most studies focus on this nucleus. It is noted that so-called exchange-NMR techniques can be used to probe ultra-slow reorientations of β(t), which in sufficiently well-defined vesicles of suitable size are dominated by the diffusion effect [40].

The relevance of intermediate motions for ^31^P spectra is illustrated in Figure 18b, where it is seen that the addition of the bolapolyphile B12 to DOPC causes significant line broadening. The overall width of the powder pattern Δν, plotted in Figure 18c, reflecting the CS anisotropy and here being related to the order parameter associated with the headgroup, does not change upon B12 addition, in agreement with results from DIPSHIFT NMR [23] focusing on $S_{\text{CH}}$ of the acyl chains. As a broadening of the underlying isochromates can also arise from sample inhomogeneity (susceptibility effects), Hahn echo experiments are required to extract transverse relaxation times $T_{2}$. A series of static spectra with increasing echo delay *τ*, see Figure 18a, can be evaluated at different spectral frequencies associated with different orientations 〈β〉. $T_{2}$ is generally a function of 〈β〉, and is predicted to be minimal at an average orientation 〈β〉 = 45° [36,111]. At this angle, $P_{2}\left(\cos\beta \right)$ has its steepest slope, which imparts the highest sensitivity on small-angle motions. The data in Figure 18d confirm that, indeed, the addition of B12 leads to shorter $T_{2}$ and thus appears to induce a larger amplitude or a decrease of the average correlation time of intermediate motions, the latter suggesting a softening of the membrane [36].

It is noted that the decrease of $T_{2}$ with temperature suggests that the “motional-narrowing” condition (which in simplest terms implies an increase of $T_{2}$ upon heating for a process with a single, thermally activated correlation time), enabling an analysis on the basis of standard relaxation theory [36], does not readily apply. It appears that a re-consideration of several sets of published $T_{2}$ data [111] in terms of a suitable slow-motional theory [113] may be on order.

Extracting meaningful quantities, with the membrane bending rigidity (more specifically, the splay elastic constant) being the usual target quantity, from $T_{2}$ data is a challenge in many regards [111,112]. Thermally activated membrane undulations are also dependent on mechanical anisotropy, the effective viscosity and possibly on hydrodynamic membrane–membrane interactions in stacks of lamellae as found in MLVs [112]. Importantly, they cover a spectrum of correlation times related to modes with different wavelengths, and low and high mode number cut-offs need to be assumed. As noted above, ambiguities in the specific relaxation theory to be used have apparently not been fully resolved.

Given the large number of parameters, a single $T_{2}$ value for a given orientation hardly suffices to draw any specific conclusion. This is why $T_{2}$ relaxation dispersions taken from Carr–Purcell–Meiboom–Gill (CPMG) experiments (or the quadrupolar analogue) have been in the focus [36,111,112,113]. The pulse sequence in Figure 18a shows the principle, where *n* is varied to measure a single $T_{2}$ decay curve, and the repetition delay 2τ is varied independently to realize different effective (angular) pulse repetition frequencies ω=π/τ. The quantity $T_{2}\left(\omega ,\langle \beta \rangle \right)$ enables more meaningful fits to theory, but holds its specific challenges.

On the technical side, such experiments are time-consuming, as many spectra after 180° pulse trains of variable length *n* need to be recorded in order to extract the orientation dependence, not to speak of *ω* variation. Alternatively, single-point acquisition of echo-top intensities during the echo train is customary for CPMG experiments, but leads to a strongly non-exponential (stretched) decay due to the $T_{2}$ anisotropy, which can only be deconvoluted on the basis of an assumption concerning the anisotropy $T_{2}\left(\beta \right)$, which in turn has been criticized [112]. Moreover, the multiple 180° pulses may interfere with the necessary ^1^H heteronuclear decoupling leading to unwanted coherent and polarization-transfer effects, and both irradiations may lead to sample heating at long relaxation delays and high *ω*.

**Figure 19:**
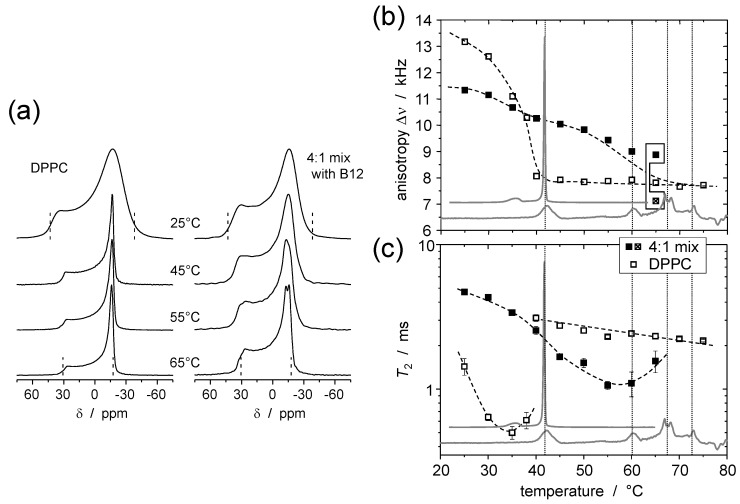
(**a**) Static ^31^P spectra of MLVs of DPPC, pure and as a 4:1 mixture with B12 taken at different temperatures. The dashed lines highlight the apparent spectral narrowing on going from the gel to the fluid $L_{\alpha }$ phase; (**b**) Temperature-dependent width of the spectra $\Delta \nu =\frac{3}{2}\delta \omega_{0}/2\pi$, see Equation (2); and (**c**) temperature-dependent $T_{2}$ relaxation times from Hahn-echo experiments evaluated for 〈β〉≈ 55°. DSC traces are shown in the background in grey. Figure reproduced with permission from ref. [22]. Copyright 2015 John Wiley & Sons.

All this considered, we have limited our investigations to studying ^31^P spectra and Hahn-echo $T_{2}$ to check the influence of bolapolyphile (B12) addition. It is noted that the Hahn-echo $T_{2}$, even for a single orientation 〈β〉, is predicted to be non-exponential in the slow-motion regime [113], challenging the definition of a meaningful $T_{2}$ value. However, this effect was found to be small, justifying a force-fit to a single exponential. Data for DPPC, in which B12 forms phase-separated structures, are collected in Figure 19. The data for pure DPPC nicely demonstrates the features of the transition from the gel phase, characterized by a rather broad spectrum, to the fluid $L_{\alpha }$ phase. In the gel phase, a $T_{2}$ minimum is observed, at which rotations around the lipid long axis cover the transition from the slow to the fast (narrowing) regime before it increases discontinuously at the phase transition.

B12 addition changes the situation qualitatively. We remind that in the context of Figure 4, it was found that only around 10% of the DPPC in the 4:1 mixture exhibits bulk behavior, while the rest is incorporated into a supramolecular structure. The anisotropy values plotted in Figure 19b demonstrate that the headgroup is apparently more mobile in this composite structure than in the gel phase, but more restricted than in the fluid phase. The anisotropy, as well as $T_{2}$ plotted in Figure 19c, is a strong function of temperature and reaches values comparable to the bulk phase only beyond 65 °C. Only at that temperature was the spectrum sufficiently well resolved to identify the two coexisting components, just before the supramolecular structure dissolves.

## 6. Conclusions

In this review, we have given an overview of traditional and advanced solid-state NMR (SSNMR) techniques that are of use in characterizing structural and dynamic features in lipid membranes and related composite systems. Specifically, the discussed examples cover work from a DFG-funded Research Unit, which has focused on the interaction of polyphilic polymers and small molecules with lipid model membranes. The main impetus was to elucidate the molecular underpinnings of macroscopic properties (thermodynamics, phase-separated superstructures) by techniques allowing for a spectral distinction between the lipid and the guest molecules.

To this end, high-resolution SSNMR techniques using magic-angle spinning (MAS) are the best choice. ^1^H spectra of soft lipid systems are sufficiently well resolved even at moderate spinning frequencies of 5–10 kHz, enabling the evaluation of simple spectral integrals representing the components that are part of fluid lipid phases. ^13^C MAS NMR comparing different excitation schemes can be used to obtain still qualitative but much more detailed information on the timescale of molecular dynamics and motional anisotropy in different lipid phases.

Both ^1^H and ^13^C MAS NMR can be extended into a second spectral (or just time) dimension for a quantitative assessment of motion-averaged (residual) homo- and heteronuclear dipolar coupling constants, respectively. These can be converted to dynamic order parameters reporting on the amplitude of fast anisotropic orientation fluctuations as constrained by the liquid-crystalline phase. For ^13^C, we have discussed in detail the respective potentials and advantages of the so-called DIPSHIFT and R-PDLF experiments, which provide carbon- and proton-detected local field measures of the ^1^H–^13^C dipolar coupling, respectively. As for ^1^H homonuclear dipolar couplings, ^1^H double-quantum MAS NMR enables the study of rather weak order parameters of minority species in the membrane at comparably high dilution.

In the final section, we have presented a survey of static ^31^P NMR experiments providing a direct probe of lipid headgroup dynamics. Spectral lineshapes, as well as $T_{2}$ relation times, provide direct means of observing the effect of guest molecules on the headgroup dynamics. In fluid ($L_{\alpha }$) phases ^31^P, $T_{2}$ is dominated by undulations of the membrane, which in turn depend on its viscoelastic properties. The potential and some of the limitations of more related quantitative studies have been discussed.
